# Coumarins and Gastrointestinal Cancer: A New Therapeutic Option?

**DOI:** 10.3389/fonc.2021.752784

**Published:** 2021-10-11

**Authors:** Zarrin Banikazemi, Seyed Mohammad Mirazimi, Fatemeh Dashti, Mohammad Reza Mazandaranian, Maryam Akbari, Korosh Morshedi, Fatemeh Aslanbeigi, Amir Rashidian, Mohsen Chamanara, Michael R. Hamblin, Mohsen Taghizadeh, Hamed Mirzaei

**Affiliations:** ^1^ Research Center for Biochemistry and Nutrition in Metabolic Diseases, Institute for Basic Sciences, Kashan University of Medical Sciences, Kashan, Iran; ^2^ Student Research Committee, Kashan University of Medical Sciences, Kashan, Iran; ^3^ School of Medicine, Kashan University of Medical Sciences, Kashan, Iran; ^4^ Department of Community Nutrition, School of Nutritional Sciences and Dietetics, Tehran University of Medical Sciences, Tehran, Iran; ^5^ Department of Surgery, Kashan University of Medical Sciences, Kashan, Iran; ^6^ Department of Pharmacology, School of Medicine, Aja University of Medical Sciences, Tehran, Iran; ^7^ Toxicology Research Center, Aja University of Medical Sciences, Tehran, Iran; ^8^ Laser Research Centre, Faculty of Health Science, University of Johannesburg, Doornfontein, South Africa

**Keywords:** Coumarin – Benzimidazole, therapy, gastrointestinal cancer, natural compound, gastric cancer

## Abstract

Cancers of the gastrointestinal (GI) tract are often life-threatening malignancies, which can be a severe burden to the health care system. Globally, the mortality rate from gastrointestinal tumors has been increasing due to the lack of adequate diagnostic, prognostic, and therapeutic measures to combat these tumors. Coumarin is a natural product with remarkable antitumor activity, and it is widely found in various natural plant sources. Researchers have explored coumarin and its related derivatives to investigate their antitumor activity, and the potential molecular mechanisms involved. These mechanisms include hormone antagonists, alkylating agents, inhibitors of angiogenesis, inhibitors of topoisomerase, inducers of apoptosis, agents with antimitotic activity, telomerase inhibitors, inhibitors of human carbonic anhydrase, as well as other potential mechanisms. Consequently, drug design and discovery scientists and medicinal chemists have collaborated to identify new coumarin-related agents in order to produce more effective antitumor drugs against GI cancers. Herein, we summarize the therapeutic effects of coumarin and its derivatives against GI cancer.

## Introduction

Cancer accounts for about 13% of all deaths globally (~7.9 million per year) and is considered a major cause of mortality (1). It has been projected that the cancer death toll will increase to 12 million by 2030 (2). Tumors of the gastrointestinal (GI) tract are the second most frequent cause of cancer-related mortality worldwide (3). According to studies performed in 2008, GI tumors are the fourth and fifth most frequent type of malignancy in males and females, respectively (4). The major GI cancers include tumors of the esophagus, stomach, pancreas, liver, and colon (5). Genetic heterogeneity in several genes, including tumor-promotor genes, mismatch repair genes, and anti-oncogenes, can all contribute to the tumorigenesis in the GI tract (6).

Moreover, an imbalance between cellular apoptosis and cellular proliferation can result in the development of GI tumors (7). Carcinogenesis of the GI tract is influenced by many intrinsic and external risk factors, including alcohol use, being overweight, genetic mutations, as well as infection with specific bacteria such as *Helicobacter pylori* (8). Most patients with GI tumors report symptoms only after the lesion has progressed, and invades into either adjacent or distant organs. Nevertheless, the most common symptoms are epigastric pain, upper abdominal bloating, and a palpable abdominal mass (9). Altogether, these tumors put a pressing load on health care providers and are considered a public health issue (8). Patients with GI tumors can have widely different prognoses, depending on the tumor type and the stage at diagnosis. The results of most patients with GI tumors can be improved with early diagnosis. Surgical resection of the tumor, chemotherapy regimens including mitomycin, cisplatin, or docetaxel, and radiotherapy alone or combined with chemotherapy are the most common treatment approaches in patients with GI cancer (10).

Phytochemicals are a group of non-nutrient bioactive compounds, widely found in many plants such as grains, vegetables, and fruits. Phytochemicals are often used to protect against the development of chronic disorders, such as cancer, neurodegenerative disorders, and cardiovascular disease (10, 11). Various studies have evaluated the chemopreventive activity of phytochemicals in GI tumors, and have shed light on their potential protective mechanisms against cancer (2).

Coumarin is a benzopyranone compound with diverse functions (12). Coumarin is usually extracted from naturally occurring sources, as well as being prepared by chemical synthesis. Coumarins are typically found in various plants, such as rhizomes, bark, leaves, plant roots, and even in marine plants (12).

Coumarins can exert a wide range of pharmaceutical functions, including activity against bacteria, viral infections, and fungal infections. It also has anti-cancer, anti-inflammatory, anticoagulant, and anti-hypertensive activity (13, 14). The benzopyrone backbone of coumarin contains many different possible substitution regions. The possible variations in the basic coumarin structure can be divided into five distinct classes: simple coumarins, isocoumarins, furocoumarins, pyranocoumarins, and dicoumarins. Laboratory researchers have produced a wide range of more complicated coumarin–derived structures, with wider applicability and more powerful functionality. Coumarin and its derivatives have shown a variety of therapeutic benefits in cancer patients. Coumarin exerts its anti-cancer effects through several mechanisms of action, such as suppression of CA (carbonic anhydrase) activity, suppressing MDR (multiple drug resistance), promoting cell apoptosis, and increased activity of the PI3K/Akt/mTOR signaling pathway (15). Herein, we summarize some therapeutic effects of coumarin against GI cancer.

## Pharmacokinetics of Coumarin

When coumarins are given orally, they are immediately absorbed through the mucosa of the GI tract and then disseminated throughout the entire body (16). Both coumarin and its derivative 7-hydroxycoumarin are water-insoluble. Nevertheless, these compounds still have a significant distribution coefficient. Coumarins can rapidly cross the lipid bilayer of the plasma membrane *via* passive diffusion, because they have a nonpolar structure (17). Studies in clinical pharmacokinetics have shown that coumarins are mainly metabolized in the liver *via* the first-pass effect, and only 2–6% of the absorbed coumarin enters the peripheral circulation (16).

Coumarin has a relatively short half-life and poor bioavailability. Pharmacokinetics studies have shown that approximately 35% of coumarin is bound to plasma proteins, whereas 47% of 7-hydroxycoumarin is bound to plasma proteins (16, 18–21). Coumarin undergoes an initial metabolic process in liver microsomes mediated by CYP2A6 (cytochrome P-450-bound mono-oxygenase enzyme), which results in the production of 7-hydroxycoumarin (22, 23). Phase-II conjugation results in the production of a glucuronide conjugate from 7-hydroxycoumarin (16, 24, 25). Coumarin and its related metabolites are readily excreted *via* the urine (26–29).

Hemorrhage is considered to be the main risk of coumarin administration, due to its anticoagulant effects. Administration of oral anticoagulants for prolonged periods is associated with an increased risk of bleeding (30, 31). Warfarin is a coumarin frequently used as an anticoagulant, and can cause unexpected bleeding, especially in female patients, with duration of therapy of 4 months or longer within the previous year, and those with an advanced age (31). As an anticoagulant, coumarin suppresses the metabolism of vitamin-K-dependent coagulation pathways, leading to the defective metabolism of bone minerals. This defective bone metabolism occurs mainly in older patients, and postmenopausal women who receive chronic administration of anticoagulants (30, 32). Small blood vessels such as venules and arterioles can undergo acute thrombotic events following consumption of oral anticoagulants such as coumarin; this can result complete skin necrosis, particularly in the thighs, lower extremities, and breasts. Administration of coumarin-based oral anticoagulants during pregnancy carries a higher risk of miscarriage. Consumption of warfarin between the 6^th^-12^th^ weeks of gestation can lead to warfarin embryopathy, which is the most severe adverse effect of warfarin. Warfarin embryopathy includes fetal abnormalities such as narrowing of the upper respiratory tract, hypoplasia of the nasal bone, and epiphyseal stippling (30–33).

## Coumarin: Structure and Anti-Cancer Activity

Coumarins are currently categorized into four distinct classes: pyranocoumarins, furanocoumarines, simple coumarins, and coumarins with pyrone-substituents (34). Simple coumarins include alkoxylated, alkylated, and hydroxylated–derivatives of coumarin, and associated glycosides, such as skimmin, umbelliferone, esculetin, herniarin, limettin, esculin, daphnin, and daphnetin (34). Furanocoumarins contain a furan ring bound to the coumarin ring. Furanocoumarins can be categorized into two groups based on the ring fusion sites: linear furanocoumarins attached at C6/C7 and angular furanocoumarins attached at C7/C8. Psoralen, imperatorin, and xanthotoxin are linear furanocoumarines, while bergapten, isobergapten, pimpinellin, isopimpinellin, and angelicin are examples of angular furanocoumarins (34–37). Pyranocoumarins have a 6-membered pyran ring attached to the benzene ring at C7–8 (angular) or C6-7 (linear). Seselin, visnadin, and xanthyletin are examples of pyranocoumarins (38, 39). Coumarins with pyrone-substituents are divided into three different groups: 3-phenylcoumarin (gravelliferone and coumestrol); 4-hydroxycoumarin (icumarol and novobiocin), and 3,4-benzocoumarin (alternariol). Plants do not contain 4-hydroxycoumarins in their natural state. Warfarin is also a synthetic compound belonging to this family (36, 40).

Coumarin compounds with multiple biological targets have been recently identified, and these could be used as new therapeutic agents to treat disorders, such as congestive heart failure and cancer. Naturally occurring drugs have become popular, since they are relatively cheap, have low toxicity, do not cause the development of resistance, as well as having significant efficacy (41, 42). Consequently, novel compounds extracted from plants and microorganisms can be combined with current chemotherapeutic drugs for cancer treatment (43). Coumarins are a large family of natural agents with diverse pharmacological properties. These compounds are currently extracted from a wide range of plants, such as *Artemisia*, *Achillea*, and *Fraxinus* genera, but they can also be synthesized in the laboratory using standard chemical reactions. Various techniques, including reflux, maceration, ultrasonic-mediated, and microwaves, have been used to extract and purify coumarin derivatives from plant source material. In the laboratory, organic reactions such as Von Pechmann, Perkin, Wittig, and Knoevenagel have been used to synthesize coumarins (42). The shikimic acid pathway plays a pivotal role in coumarin biosynthesis in nature. The shikimic pathway consists of a series of enzymatic reactions resulting in the production of umbelliferone, chorismic acid, p-coumaric acid, and cinnamic acid. In addition, the enzyme cytochrome P450 plays a major role in converting cinnamic acid into isofraxidin, umbelliferone and scopoletin through an ortho-hydroxylation reaction (42, 44).

It is well known that many cancers can recur after being treated with conventional chemotherapy. This phenomenon is known as multidrug resistance (MDR), which is often due to up-regulation of transmembrane protein drug-efflux pumps, including p-glycoprotein (P-gp) also known as ATP-binding cassette sub-family B member 1 (ABCB1), or multidrug resistance-associated protein 2 (MRP2, ABCC2) which can actively pump many anti-cancer drugs out of the cells, a process powered by ATP hydrolysis. In this context, coumarins have the potential to decrease the activity of MRP2 and P-gp, and could overcome MDR.

Baghdadi et al. (45) isolated six coumarin derivatives, including mansorin-I, mansorin-II, mansorin-III, mansorin-A, mansorin-B, and mansorin-C, from *Mansonia gagei* a plant of the Sterculariaceae heartwood family. Their study showed that these agents had promising antitumor activity against hepatocellular carcinoma, breast cancer, colorectal cancer, and cervical cancer cell lines. In this context, mansorin-II and mansorin-III had the highest antitumor effect, with a half-maximal inhibitory concentration (IC50) of 3.95-35.3 µM and 0.74 -36 µM, respectively. Moreover, mansorin-II was able to potentiate the antitumor effects of taxol. This effect occurred partly by inhibiting the P-gp efflux activity.

Carbonic anhydrase is a zinc-containing metalloenzyme, responsible for catalyzing the reaction between carbon dioxide and water to produce carbonic acid, bicarbonate, and hydrogen ions. This reaction maintains the balance between the intracellular and extracellular pH at stable levels, and allows the transfer of ions through the transmembrane space, and other metabolic processes to proceed (46). Sixteen different carbonic anhydrase enzymes have been identified. Of these, CA I and CA II are cytoplasmic enzymes, while CA IX and CA XII are transmembrane proteases. Because cancer cells and their surrounding microenvironment exist in a state of hypoxia, they increase their rate of glycolysis to satisfy their metabolic requirements, and therefore lactic acid accumulates in the tumor microenvironment. CA XII and CA IX are up-regulated in tumor cells dur to the action of HIF (hypoxia-inducible transcription factor). Carbonic anhydrase enzyme plays a role in the growth and metastatic dissemination of primary tumors, as well as the development of resistance to chemotherapy (46, 47).

CA IX and CA XII are highly expressed in many cancer types, and may be promising targets for therapeutic intervention. Belma et al. (48) investigated a wide range of compounds for their inhibitory effect on CA XII, CA IX, CA II, and CA I enzymes in colorectal cancer cells. They found that some compounds could effectively inhibit both CA XII and CA IX. The most active was 4-((((2-((1-(3-((2-oxo-2H-chromen-7-yl)oxy)propyl)-1H-1,2,3-triazol-4-yl)methoxy)naphthalen-1-yl)-methylene)amino)methyl)benzenesulfonamide, which could selectively inhibit proliferation in colorectal cancer cells, and had an inhibitory constant (Ki) of about 596.6 nM for CA XII and 45.5 nM for CA IX.

The caspase family of enzymes are involved in the induction of apoptosis. These enzymes include caspase-10, 9, 8, 7, 6, 3, and 2. Among these, caspase-10, 9, 8, and 2 are involved in the initiation of apoptosis. Caspase-2 catalyzes its own cleavage and becomes activated to trigger apoptosis under the influence of intracellular signaling. Subsequently, the process of apoptosis is executed by caspase-7, -6, and -3, which are activated by upstream promoters. These caspases cleave various functional and structural proteins, especially PARP (poly-ADP-ribose polymerase) (49). Bcl-2 (B cell lymphoma-2) is a major tumor-promoter gene that generally inhibits cellular apoptosis. The Bcl-2 family contains proteins with both anti-apoptosis and pro-apoptosis activity. PUMA, Bax, and Bad are examples of pro-apoptotic proteins which are predominantly found in the cytoplasm. After being activated by apoptosis signaling, these proteins migrate to the mitochondrial outer membrane, where they form transmembrane channels that allow the mitochondria to expel cytochrome C, thus activating caspases resulting in apoptosis. On the other hand, the Bcl-2 family also contains proteins with anti-apoptotic properties, primarily found in the mitochondrial outer membrane, which can inhibit apoptosis by preventing loss of cytochrome C from the mitochondria (49). Coumarin-derived compounds can modulate the expression of pro-apoptotic proteins and could thus help to treat malignant tumors.

Nordin et al. (50) extracted a coumarin compound known as PulchrinA, from a Malaysian plant called *Enicosanthellum pulchrum* in the Annonaceae family. They evaluated the potential of this coumarin-derived agent to produce apoptosis in ovarian tumor cells. Pulchrin A was found to reduce Bcl-2 expression and increase Bax protein expression *via* increasing caspase-9 and caspase-3 activity, with an IC50 level about 22 μM in ovarian cancer cells.

The phosphatidylinositol kinase PI3K is found in the intracellular compartment. PI3K can activate other protein kinases such as PKC, PKB, and PKA, and plays a pivotal role in processes, such as cell differentiation, growth, migration and apoptosis. AKT or PKB is a serine/threonine kinase, which is a downstream target for PI3K, and is significantly correlated with cellular proliferation and apoptosis. In addition, AKT activates CDK2 and CDK4 and modulates p27, and acts as a cyclin-dependent kinase inhibitor, thereby preventing cell cycle progression. AKT also has anti-apoptotic activity by acting on several pathways. Some examples of the anti-apoptotic activity of AKT include, inhibiting caspases and Bax, inhibiting GSK3 activity (which increases apoptosis through cleavage of the cytoskeleton protein β-catenin), as well as reducing the adhesion of cells. AKT can increase the activity of transcription factor NF-κB which in turn leads to increased repair of DNA damage, reducing pro-apoptotic FasL gene expression, and inhibiting the release of cytochrome C out of the mitochondria. mTOR is another threonine/serine kinase, which is a downstream target of AKT. Following its own activation, mTOR activates the ribosomal proteins p70S6K and E-BP1 (a translation inhibitor) (4). The binding of 4E-BP1 to eIF-4E becomes weaker upon phosphorylation; as a result the free eIF-4E is able to bind to other factors to initiate protein translation.

After being activated, p70S6K can increase protein production. The PI3K/AKT/mTOR pathway plays a crucial role in the modulation of the cell cycle, cell viability, proliferation, differentiation, and metastasis (51, 52). This signaling pathway has been shown to be correlated with human carcinogenesis. Abnormal up-regulation of this signaling pathway has a role in the formation, growth, progression, and chemoresistance of cancer cells, and could be a new potential therapeutic target for cancer treatment (53, 54). 5-Methoxypsoralen is a linear furocoumarin (psoralen) extracted from plant sources (such as parsley and bergamot) by alkali treatment. In a study by Guo et al. (55), 5-methoxypsoralen was found to inhibit PI3K, mTOR, and Akt phosphorylation and expression in human glioma cells, resulting in the inhibition of the PI3K/Akt/mTOR signaling axis. Following exposure to 5-methoxypsoralen, the DNA in glioma cells was damaged by fragmentation, and abundant autophagic vacuoles were formed.

Microtubules are an important constituent of the cell cytoskeleton, and control cell cycle progression, proliferation, cell morphology, and intracellular signaling. Several anticancer drugs can cause microtubule depolymerization, or else they block microtubule aggregation, resulting in cell cycle arrest at the M-phase, and thus mitosis is blocked in tumor cells. Microtubules have three different binding sites for the anticancer drugs, vincristine, paclitaxel, and colchicine. Therefore paclitaxel, vincristine, and colchicine are often used to inhibit microtubules. Moreover, these drugs are substrates of efflux systems mediated by P-gp pumps, resulting in multidrug resistance in cancer cells. A study by Dahong evaluated the effects of the coumarin derivative, Ferulin C on proliferation in breast cancer *in vitro* and *in vivo*. Ferulin C is a coumarin isolated from the roots of *Ferula ferulaeoides*, which can also bind to colchicine binding sites on β-tubulin, thus preventing its aggregation. Ferulin C can inhibit the polymerization of tubulin (IC50 = 9.2 μM) compared to colchicine (IC50 = 1.8 μM) used as the reference. Ferulin C was found to specifically alter the microtubule structure without affecting tubulin expression. Ferulin C destablilized microtubules, and increased the activity of p21, while it suppressed PAK1. Higher levels of PAK1 are correlated with unfavorable outcomes, while higher levels of p21 are correlated with favorable outcomes in patients with breast cancer.

Ferulin C caused cell cycle arrest at the G1/S phase by activating the p21Cip1/Waf1-CDK2 signaling axis. A xenograft model of breast tumor was used in an *in vivo* study by Dahong, where they assessed the anti-cancer effects of Ferulin C (low dose, 25 mg/kg; median dose, 50 mg/kg; high dose, 100 mg/kg). Their study showed that Ferulin C could block breast tumor cell proliferation in the xenograft model, and this anti-cancer activity correlated with the *in vitro* results (56).

## Coumarins and Gastric Cancer

Gastric cancer (GC) is among the most prevalent GI tumors globally. Approximately 1 million patients are newly diagnosed with gastric carcinoma each year. Because of its aggressiveness, gastric cancer is the third cause of cancer-related death (57). However, the conventional drugs are limited by unwanted toxicity and adverse side effects due to their poor selectivity for cancer cells compared to normal mammalian cells (58). Consequently, identifying novel therapeutic agents with lower toxicity is needed for more successful management of patients with gastric cancer.

Molecular hybridization is a novel concept in drug development and discovery. It is based on combining two or more biologically active molecules by attaching them together using appropriate covalent bonds. Compared to their individual elements, the hybridized structures show superior or novel biological functions (59).

Nucleotides are nitrogen-containing heterocyclic structures that are basic components of RNA and DNA (60, 61). Because nucleobases play major roles in various cellular processes, nucleotides are often utilized as pharmacophores, especially in antitumor drugs (62–64). Interestingly, nucleobases can show *in vitro* cytotoxicity in a variety of human tumor cell lines (64) caused by several mechanisms. The click reaction is an easy synthetic approach to prepare the triazole scaffold (a nitrogen containing heterocyclic compound) frequently used as a linker in pharmaceutical research (65). Furthermore, the use of 1,2,3-triazole is associated with increased solubility (66), improved strength of binding to other biological compounds, and can show synergistic effects on biological functions (67). Considering their specific rigid structure and binding to particular hormone receptors, steroids are a major family of biological compounds, widely used in drug design (68). Modification of the C-16 atom in steroids can be used to attach other moieties, in order to produce tumor-targeted cytotoxic agents (69–73).

Using the molecular hybridization technique, Zhao et al. prepared a group of analogues of the 1,4-disubstitued 1,2,3-triazole-nucleobase, including additional moieties such as steroids, coumarins, or quinolines (74). In their study, a number of these compounds were shown to suppress the cellular proliferation of tumor cells. In this context, compound 20c showed an anti-proliferative activity in SGC-7901 cells (IC_50_ = 2.28 μM) and MGC-803 cells (IC_50_ = 1.48 μM) and did not affect healthy non-cancerous cells. Compound 20c may inhibit TGF β1 expression in gastric cancer cell lines, and suppress cellular invasion and migration. Compound 20c could be used as a novel skeleton for therapeutic agents against GC with minimal side effects (74).

ISOIM (isoimperatorin) is a member of the 6,7-furanocoumarin family and is isolated from plants in the umbelliferae family, including *Heracleum maximum*, *Angelica dahurica*, *Peucedanum ostruthium.* Chinese angelica has been frequently utilized in ancient Chinese medicine (75). Isoimperatorin is a secondary plant metabolite with numerous pharmacological properties, such as anti-hypertensive, analgesic, anti-inflammatory, antitumor, antiviral, and antibacterial activity (76–80). In addition, ISOIM can inhibit proliferation in several cancer cell lines, including skin cancer SK-MEL-2, ovarian cancer SK-OV-3, lung cancer A549, breast cancer MCF-7, glioblastoma XF498, and colon cancer HCT-15 (76–81). ISOIM was found to suppress the proliferation of SGC-7901 gastric cancer cells and modify the expression of several anti- and pro-apoptotic proteins (82).

In a study by Yang et al., the pro-apoptotic and anti-proliferation properties of ISOIM in BGC-823 gastric cancer cells were evaluated, along with the potential biological mechanisms (83). The MTT assay measured cellular proliferation, while hematoxylin and eosin staining, acridine orange/ethidium bromide staining, and Hoechst 33258 were employed to assess cell morphology. Flow cytometry assays measured apoptosis and cell cycle status, and the expression level of pro-apoptotic proteins was evaluated using Western blotting. It was found that ISOIM could suppress proliferation through inducing cell cycle arrest at the G_2_/M stage. Moreover, ISOIM induced apoptosis through increased expression of Bax (Bcl-2-associated X) and reduced expression of Bcl-2, thus reducing the Bcl-2/Bax ratio compared to the control cells. Furthermore, the administration of ISOIM led to cytochrome *c* release from the mitochondria into the cytosol, along with activation of caspase-3, indicating that apoptosis was stimulated by the mitochondrial pathway in BGC-823 cells (83).

Perumalsamy et al. performed an *in silico* and *in vitro* study to investigate whether SSBC (styrene substituted biscoumarin) could induce apoptosis and inhibit proliferation of tumor cells (84). The MTT assay was used to measure proliferation in gastric cancer (AGS) cell lines in addition to healthy lung cell lines (MRC-5 and L-132). Molecular docking was used to examine the binding between SSBC and Bcl2. Moreover, PASS (spectrum prediction analysis) was used to evaluate the biological effects, and ADME was used to measure pharmacological properties and drug likeliness. DAPI/PI staining, Hoechst staining, and FACS were employed to evaluate SSBC-induced apoptosis in AGS cells. Western blotting and Quantitative Real-Time Reverse Transcription (qRT-PCR) were used to investigate the mechanisms of apoptosis induction. The IC50 values of SSBC for MRC-5 and L-132 cells were 285 and 268 μg/mL, respectively, while for AGS cells the IC50 was 4.56 μg/mL. *In silico* analysis predicted that SSBC could bind to the BH3 domain of anti-apoptotic proteins, which could then activate apoptosis and cell death. Using ADME predicted that SSBC had a high binding affinity (~ 99.08%) and a high absorption rate (~95.57%) in the small intestine. The PASS software suggested that SSBC could affect the expression level of several proteins involved in apoptosis. Western blotting, FACS, DAPI/PI staining, qRT-PCR, and Hoechst staining confirmed apoptosis in AGS cells. SSBC may be particularly effective to trigger apoptosis mediated by the intrinsic pathway, and thus *in vivo* studies and human clinical trials for GC may be justified (84).

Farnesiferol C (FC) is a member of the coumarin family, belonging to the sesquiterpene group, which is routinely extracted from *Ferula szowitziana* DC roots (85). *Apiaceae* (genus Ferula) are plants that are widely distributed in Northern Africa, Central Asia, and the Mediterranean region (86), and these plants are a rich source of natural compounds, including coumarins and sesquiterpenes (87). FC has a variety of biological functions, including anti-tumor activity *in vitro* and *in vivo*, reducing the formation of new blood vessels, and also shows activity against Leishmania infection (88). Nevertheless, FC has poor solubility, and relatively low bioavailability both *in vivo* and *in vitro*, which hinders its potential therapeutic applications (89). However, recent studies have shown that FC solubility and antiproliferative effects against cancer cells may be improved by incorporation into dendrosome nanoparticles. Dendrosomes are spherical, covalently linked, degradable, neutral, and self-assembled nanoparticles which have become popular for their ability to deliver herbal agents and genes into various cell lines (89–93).

Aas et al. performed a study to determine the potential antitumor effects on AGS cells of DFC (dendrosomal farnesiferol C) (94). RT-PCR was used to assess the Bax/Bcl-2 ratio in order to determine apoptosis. MTT assay was used to evaluate the antiproliferative effects of DFC. DFC inhibited AGS proliferation in a time- and dose-dependent manner, compared to free FC. DFC increased the Bcl-2/Bax expression ratio in AGS cells. Taken together, the nano-formulated farnesiferol C may be useful in tumor-targeted therapy (94).

**Table 1** lists some studies describing the anti-gastric cancer effects of coumarins.

**Table 1:** Anti-gastric cancer effects of coumarins.

Coumarin compound	Dose	Mechanisms	Model	Cell line	Ref
Steroidal/coumarin/quinoline moieties	1.48 μM, 2.28 μM	Reduced expression of TGF β1, inhibited invasion and migration	*In vitro*	SGC-7901, MGC-803	(74)
Styrene substituted biscoumarin (SSBC)	4-64 μg/mL	Induced apoptosis through intrinsic pathway	*In vitro*	AGS	(84)
Isoimperatorin (ISOIM)	0.05-2 mM	Anti-proliferative and pro-apoptotic effects	*In vitro*	BGC-823	(83)
2′-Z auraptene A	1, 2, 4 μM	Anti-proliferative and pro-apoptotic effects	*In vitro*	MGC-803	(95)
Esculetin	850 μM	Inhibited proliferation, induced apoptosis through IGF-1/PI3K/Akt mitochondrial pathway	*In vitro*	MGC-803	(96)
Isocoumarin 3,4,7		Cytotoxicity	*In vitro*	MGC80	(97)
Esculetin	12.5, 25, 50 μM	**Apoptosis *via* cyclophilin D-induced mitochondrial permeability transition pore, increased ROS**	*In vitro*	GC-7901, MGC-803, BGC-82	(98)
Dendrosomal farnesiferol C (DFC)	80 μM	Inhibited proliferation, increased Bax/Bcl-2 ratio	*In vitro*	AGS	(94)
3-Bromoacetylcoumarin	IC50 = 29 nM	Significant cytotoxicity	*In vitro*	NUGC	(99)
7-Diethylamino-3(20-benzoxazolyl)-coumarin (DBC)	30, 100, 1000, 3000 nM	Induced caspase-dependent apoptosis, reduced expression of anti-apoptotic genes	*In vitro*	SNU-620, SNU- 620-5FU	(100)
Novel coumarin (compound 3d)	2.69 ± 0.60 µg/mL	Acted as telomerase inhibitor, bound to telomerase active site	*In vitro*	SGC-7901	(101)
Xanthoxyletin	200, 400 μM	Increased ROS	*In vitro*	SGC-7901	(102)
6,7-Dihydroxy derivative	100, 400 μM	Antiproliferative activity	*In vitro*	TGBC11TKB	(103)
Coumarin	—————	Quenched organic hydroperoxides and hydrogen peroxide	*In vitro*		(104)
Coumarin (C), 7-hydroxy-coumarin (7-OH-C)	IC50 = 1.59-3.57 mM for C, 0.68-2.69 mM for 7-OH-C	Inhibited cell proliferation	*In vitro*		(105)

## Coumarins and Colon Cancer

Colon cancer is a leading cause of morbidity and mortality throughout the world (106). It has the second highest incidence and cause of cancer-associated death. More than 1.8 million new cases and 881,000 deaths occurred in 2018 globally (57). The mortality of colon cancer has fallen over recent decades due to advances in both treatment and early detection; however, the five-year survival rates remain low at advanced or metastatic stages (106). At an early stage, the 5-year survival rate is nearly 90%, but the survival rate falls to near 10% when the disease is advanced. Therefore, better understanding of the molecular pathway of colon cancer progression is a serious need (107).

HMGA2 (high mobility group AT-hook 2) belongs to the high mobility group family of proteins. It is a non-histone chromatin protein with three AT-hook domains capable of binding in the minor groove of AT-rich DNA sequences (108). HMGA2 acts as an architectural transcription factor due to its ability to assemble the nucleoprotein structure leading either to transcription enhancement or repression (109). The expression of HMGA2 is higher during embryogenesis, whereas in adult tissues its expression is zero or very low (110). HMGA2 has an important function in metastasis and regulates the epithelial-mesenchymal transition (EMT) (107). The EMT is where epithelial cells transform into mesenchymal cells, and is essential for both tumor progression and embryonic development. The EMT results in elevated invasion, migration, or unrestricted proliferation (111, 112). Besides, HMGA2 is commonly upregulated in many types of cancer, and was correlated with poor prognosis and lower survival rates in colon cancer (113).

Oxidative stress modulates many cellular processes, and provides a favorable environment for cancer cells to progress and survive. Many studies have shown the importance of oxidative stress or oxidative damage in cancer initiation and progression (114). Furthermore, oxidative stress- resistance is a critical adaptive response that allows cancer cells to develop resistance to chemotherapy drugs, resulting in chemoresistance and cancer recurrence (115, 116). On the other hand, several anti-cancer treatments are based on the production of excessive reactive oxygen species (ROS) or the abrogation of antioxidant pathways to kill cancer cells (117–120).

Chen et al. studied the involvement of HMGA2 in the modulation of oxidative stress using luciferase reporter assays (121). In addition, they studied dicoumarol (DIC), a coumarin derivative involved in redox modulation that has some anti-cancer effects. It was found that DIC could trigger apoptosis and inhibit the migration of colon cancer cells that over-expressed HMGA2. DIC could also promote the anti-cancer effect of 5-FU in colony formation assays. Overall, their findings provided a novel understanding of the molecular function of HMGA2, and suggested a possible therapeutic application of DIC to prevent progression in colon cancer cells that over-express HMGA2 (121).

Sulfonamides act as inhibitors of carbonic anydrase (CA) enzymes, and show antitumor, antiepileptic, antiglaucoma, and diuretic properties (122–127). In addition, coumarin sulfonamides, and their derivatives act as selective inhibitors for two carbonic anhydrase isoforms, including CA XII and CA IX (128–132).

Zengin Kurt and his colleagues synthesized and characterized 27 novel compounds divided into three series: (1) sulfonamide-based imines (6a-6i); (2) coumarin-based aldehydes (7a-7i); and (3) coumarin-sulfonamide hybrid molecules (8a-8i), which were characterized by IR, NMR, etc. (48). These compounds were tested on different CA isoforms, including CA (I, II, IX, XII), to measure the degree of inhibition. 4-((((2-((1-(3-((2-oxo-2H-chromen-7-yl)oxy)propyl)-1H-1,2,3-triazol-4-yl)methoxy)naphthalen-1-yl)-methylene)amino)methyl)benzenesulfonamide (**8i**) was the best CA IX inhibitor with a Ki of 45.5 nM. Furthermore, 8i could also selectively inhibit colon cancer cell (HT-29) proliferation by directly targeting CA XII and CA IX (48).

Cancer treatment is affected by genetic mutations occurring in the tumor, and the survival rate in advanced or metastatic stages remains poor. For instance, one of the clinical challenges for EGFR targeted therapies is the KRAS gene mutation, so alternative approaches are needed to reduce failure in colon cancer therapy. One of these approaches may involve coumarin or its derivatives (biological or synthetic). Lin et al. synthesized five coumarin derivatives; nitro-substituted, dimethoxy-substituted, and trifluoromethyl-substituted at various locations (133). According to their findings, one nitro-coumarin derivative, 5,7-dimethoxy-4-methyl-6-nitro-chromen-2-one, had the highest cytotoxicity against colon cancer cells and triggered apoptosis. This compound could also inhibit long-term and short-term proliferation, and reduced colon cancer cell migration. It was effective against colon cancer cells with either mutant or wild-type KRAS genes (133).

**Table 2** lists some studies on the anti-colon cancer effects of coumarins.

**Table 2:** Anti-colon cancer effects of coumarins.

Coumarin compound	Dose	Mechanisms	Model	Cell line	Ref
Furanocoumarin	100 μM	Anti cancer effects	*In vitro*	HCT116	(134)
Dicoumarol (DIC)	10 μM	Inhibited proliferation in HMGA2 overexpressing cells	*In vitro*	DLD-1	(121)
Butyrate/GC combination	10 μM GC + 2mM butyrate	Increased apoptosis	*In vitro*	HCT116	(135)
Coumarin 8a		Inhibited proliferation by targeting CA XII and CA IX	*In vitro, in vivo*	HT-29	(48)
Coumarin derivatives	———	Inhibited proliferation	*In vitro*	LoVo	(136)
Copper-redox cycling by coumarin-di(2-picolyl)amine hybrid molecule	50 µM	Pro-oxidant, inhibited proliferation.	*In vitro*	HCT116	(137)
Nitro-coumarin derivative, 5,7-dimethoxy-4-methyl-6-nitro-chromen-2-one	5, 10, 20 µM	Activated apoptosis pathways	*In vitro*	HCT116	(133)
Poly(DGU-BDT) nanoparticles	100 µg/mL	Cytotoxicity, high cellular uptake	*In vitro*	HeLa	(138)
Coumarin derivatives (6, 11, 13, 19, 21, 25, 39)	30, 100, 300 µM	Inhibited CA IX and XII in hypoxia, another unidentified target in normoxia	*In vitro*	HT-29	(139)
Coumarin	IC50: >200µM	Cytotoxicity	*In vitro*	HCT116	(140)
Coumarin-6-sulfonamide derivatives (4a, b, 8a–d, 11a–d, 13a, b, and 15a–c)	8a (IC50 ¼ 8.5 3 ± 0.72)- 11a (IC50 ¼ 10.12 ± 0.90)-8d 16.02 ± 1.32- 11d 16.06 ± 1.28	Inhibited proliferation	*In vitro*	Caco-2	(141)
Coumarin-6 (TK-MS/DOX)	IC50 = 1.6 ± 0.48 mg/mL	Cytotoxicity	*In vitro*	Caco-2	(142)
Columbianadin (CBN)	25, 50 µM	Induced apoptosis and necroptosis	*In vitro*	HCT-116	(143)
coumarin Compounds 2, 3–15, 17–18, and 20–23	50 µM	Cytotoxicity	*In vitro*	HCT-116	(144)
Coumarin compound 7a	IC50 = 4.8 ± 0.18 µg.mL-1	Cytotoxicity	*In vitro*	HCT-116	(145)
Coumarins (clausarin, dentatin, nordentatin, xanthoxyletin)	————	Cytotoxicity	*In vitro*	HCT-116	(146)
Coumarins (mammeisin, mammein)	COLO205 (9.7-10.7 μM) KM12 (10.9-12.0 μM),	Inhibited proliferation	*In vitro*	COLO205, KM12	(147)
Fused tricyclic coumarin sulfonate derivatives	IC50 = 532 nM	Inhibited cyclooxygenase-2 (COX-2) enzyme	*In vitro*	HT-29	(148)
5,7-Dihydroxy-4- methyl-6-(3-methylbutanoyl)-coumarin (DMAC)	40, 80 µM	Caused cell death, PARP cleavage, activated caspase-3	*In vitro*	HCT116 and LoVo	(149)
Coumarin compounds	IC50 = 0.01- 2.8 µM	Cytotoxicity	*In vitro*	HCT-116	(150)
Coumarin compounds	————–	Cytotoxicity	*In vitro*	HT-29	(151)
Esculetin	IC50 = 55 µg/mL	Induced apoptosis, activated mitogen-activated protein kinases; specific inhibitors of these kinases protected cells	*In vitro*	HT-29	(152)
3-(1H-benzo[d]imidazol-2-yl)-7-(substituted amino)- 2H-chromen-2-one (Compound 8)	10 µM	Anticancer activity	*In vitro*	HCT-116, HCT-15	(153)
Aesculetin	20,40,80 µM	Inhibited proliferation	*In vitro*	HCT116, HCT15, DLD1	(154)
SN38-P-II, SN38-P-IV	40 mg/kg IC50 = 15 to 90 ng/mL	Anti-tumor activity	*In vivo*, *in vitro*	HT-29, HCT116	(155)
Furo[3,2-c]coumarin derivatives	IC50 = 9 µM, 8 µM	Inhibited proliferation	*In vitro*	HCT-15	(156)
Esculetin	—	Induced apoptosis (ER stress response)	*In vitro*	HT-29	(157)
(3E)-3-(4-Methylbenzylidene)-3,4-dihydro-2*H*-chromen-2-one (MBDC)	IC50 = 15.6 μg/ml	Cytotoxicity	*In vitro*	HT-29	(158)

### Coumarins and Pancreatic Cancer

More than 300,000 patients die from pancreatic cancer every year (159, 160). Pancreatic ductal adenocarcinoma (PDAC) is the most lethal and aggressive type of pancreatic cancer with less than 5% of patients surviving for five years (161). The CMGC kinome group includes tyrosine-(Y)-phosphorylation-regulated kinases (DYRKs), which carry out serine/threonine phosphorylation and tyrosine phosphorylation (162). Due to its location on human chromosome 21q22.2, which covers the Down syndrome critical region (DSCR), DYRK1A has received much interest amongst other DYRKs (163, 164). DYRK1A has been described as a double-edged kinase, because it can either act as a tumor suppressor or as an oncogene depending on the substrates and cellular environment (163). DYRK1A over-expression leads to cell cycle disturbance, cancer progression, and increased aggressiveness, so that DYRK1A could be an appealing drug target in chemotherapy. Over-expression of DYRK1A disrupts the cell cycle by phosphorylating components of the cell cycle machinery, as well as increasing the expression of Bcl-XL (anti-apoptotic protein) and phosphorylating caspase 9 at threonine residue 125 (165–167). A recent study revealed that DYRKY1A kinase could play an oncogene role in NSCLC (non-small-cell lung cancer) (113), glioma, and myeloid leukemia (108). Reducing the activity of DYRK1A in cancer cells might be a new way to attack cancers that have developed an innate resistance to pro-apoptotic stimuli (165). For some time, the involvement of DYRK1A in PDAC was not understood (168). Recently, it was discovered that DYRK1A is elevated in PDAC and has a pro-tumorigenic effect. Moreover, the expression of DYRK1A at protein and gene levels in human pancreatic tumors are both significantly increased (168). Upregulation of c-MET is another frequent alteration in PDAC. Moreover, Sprouty2 (SPRY2) is a DYRK1A substrate, which upregulates c-MET, and might increase oncogenesis (168).

Eight different isopentenyl-substituted compounds were isolated from *Glycyrrhiza uralensis* Fisch. These were, four coumarins, three flavonoids, and one benzofuran (169). Licocoumarone (LC), one of the isolated compounds, inhibited DYRK1A with an IC50 value of 12.56 μM. According to molecular docking studies, LC filled the DYRK1A pocket and generated hydrophobic contacts and hydrogen bonds with the DYRK1A amino acids. The binding of LC to DYRK1A was confirmed using drug affinity responsive target stability (DARTS) and microscale thermophoresis (MST) approaches. LC showed cytotoxicity against BxPC-3 cells that over-expressed DYRK1A with an IC50 of 50.77 μM. LC also lowered the quantity of c-MET protein, and could play a role in new pancreatic cancer treatments (169).

Fu et al. prepared paclitaxel PEG-PLGA nanoparticles emulsified in d-alpha tocopheryl polyethylene glycol 1000 succinate (TPGS) called PTX-PEG-PLGA-NP, and evaluated its effects on apoptosis in pancreatic cancer cell line (MIAPACA-2) (170). PTX-PEG-PLGA-NP was prepared in a single step utilizing TPGS as an emulsifier, and had a high drug loading. The physical and chemical properties, such as *in vitro* release and stability, were assessed, and the drug loading and particle size were used to optimize the formulation. The cellular uptake of fluorescein coumarin 6 (C6) loaded PEG-PLGA-NP by MIAPACA-2 cells was visualized using fluorescence microscopy, and flow cytometry, The MTT assay was used to measure proliferation and apoptosis of MIAPACA-2 cells following exposure to PTX-PEG-PLGA-NP. The nanoparticles had a 90.26% PTX entrapment efficiency, a 10.13% PTX loading, an average particle size of 92.33 nm, and a zeta potential of +10.48 mV. The nano preparation showed 25.9% drug release in 4 hours compared to 98.5% for Taxol for injection, thus showing a sustained-release effect. Cell uptake tests revealed that MIAPACA-2 cells steadily took up c6-PEG-PLGA-NP over time. The inhibition of MIAPACA-2 cell proliferation was not significantly different in the PTX-PEG-PLGA-NP group compared to the PTX group, according to MTT data. Flow cytometry revealed that PTX-PEG-PLGA-NP caused more apoptosis in MIAPACA-2 cells compared to PTX. The TPGS emulsification process is simple to use and environmentally friendly. The nanoparticles might be employed for pancreatic cancer treatment in the future.

**Table 3** lists some studies on the anti-pancreatic cancer effects of coumarins.

**Table 3:** Anti-pancreatic cancer effects of coumarins.

Coumarin compound	Dose	Mechanisms	Model	Cell line	Ref
Licocoumarone	25 μM, 50 μM	Inhibited DYRK1A and cell proliferation, induced apoptosis	*in vitro*	Human PDAC cell line BxPC-3	(169)
Scopoletin (SPL)	Animals: 1 mg/kg bw/day	Decreased activating transcription factor 6 (ATF6) and protein kinase RNA-like endoplasmic reticulum kinase (PERK) in β-cells**;** reduced expression of eukaryotic initiation factor2α, X-box binding protein 1, C/EBP homologous protein, ATF4	*in vitro in vivo*	Rat insulinoma 5f (RIN5f) cells, Sprague Dawley rats	(171)
Coumarin– triazole			*in vitro*	Porcine pancreatic lipase (PPL)	(172)
Furocoumarin (psoralen**)**		Selective cytoroxicity attributed to inhibition of mtKv1.3	*in vitro*	B16F10 cells	(173)
Isoprenylated coumarins	25 μM	Compound 6 showed highest cytotoxicity with an LC50 of 4 µM. induced apoptosis following a 24-h incubation	*in vitro*	Panc-1	(174)
Bromocoumarin hydrazide-hydrazone derivative (BCHHD)	50 μM	Induced apoptosis by caspase 3/7 activation, inhibited CYP3A4 and GST in a dose-dependent manner, induced cytotoxicity	*in vitro*	Panc-1	(175)
Daphnetin (7, 8-dihydroxycoumarin)	1, 10, 20, 40 μM)	Pre-treatment with daphnetin increased glucose stimulated insulin secretion, decreased lipid peroxidation markers, increased antioxidant enzymes in STZ-induced INS-1 cells.Inhibited apoptosis by increasing Bcl-2 protein, down-regulated Bax and NF-κB protein levels	*in vitro*	INS-1	(176)
Derivatives of 3‐arylcoumarin (6a‐f and 7a‐f)	(0‐100 μM)CC-50		*in vitro*	(BxPC3)	(177)
Fluorescein coumarin 6 (C6)		Inhibited proliferation, induced apoptosis	*in vitro*	MIAPACA-2	(170)
Geranylgeranyl ether coumarin derivative 9	6.25 lM	Induced apoptosis and morphological changes within 24 h	*in vitro*	PANC-1	(178)
Esculetin	100 μM	Inhibited proliferation, cell cycle arrest at G1-phase, induced mitochondrial apoptosis, activated caspases 3, 8 and 9. Lowered intracellular ROS, decreased protein levels of p65-NF-κB	*in vitro*	PANC-1, MIA PaCa-2, AsPC-1	(179)
FuranocoumarinBergamottin	Bergamottin 25 μM.	Induced membrane blebbing, cell shrinkage, organelle disintegration in PANC-1 cells. Inhibited PANC-1 migration and colony formation. Bergamottin inhibited Akt/mTOR signaling pathway.	*in vitro*	PANC-1, MIA PaCa-2, PSN-1	(180)
4-Methylumbelliferone (MU)	0–100 µM	7-Hydroxy group was critical for inhibition of HA synthesis, two hydroxy groups, 5,7 or 6,7 positions, were more effective	*in vitro*	KP1-NL	(181)
17 synthetic coumarins	100 μM	Six compounds were shown to have poor activity against PANC-1. Two trifluoromethylphenyl compounds **33** and **34** were effective against three cancer cell lines, The position of the trifluoromethyl substituent on the phenyl ring *(meta vs. para)* was associated with selective activity against MIA and PaCa-2 cells	*in vitro*	PANC-1, MIA PaCa-2, Capan-1	(182)
7-Hydroxy-2-oxo-2*H*-chromene-3-carboxylic acid (3-phenylpropyl)amide (**2c**)	PC50 = 0.44 μM	Inhibited PANC-1 colony formation and migration in a concentration-dependent manner, compound **2c** was lead structure against pancreatic cancer.	*in vitro*	PANC-1	(183)
Derivatives of 6-brominated coumarin hydrazide-hydrazone (BCHHDs)	IC50: 3.60-6.50 μM	Activated caspase 3/7, induced apoptosis in resistant Panc-1 cells. Microarray analysis showed that BCHHD **7c** induced apoptosis and cell cycle arrest (G2/M), and up-regulated CDKN1A, DDIT4, GDF-15 genes, and down-regulated CDC2, CDC20, CDK2 genes	*in vitro*	Panc-1	(184)
Daphnetin	4 mg/kg body weight IP 30 min before the injection of sodium taurocholate.	Daphnetin decreased serum alanine transaminase and creatinine (CR) levels, increased superoxide dismutase (SOD) activity, lowered apoptosis and neutrophil infiltration of pancreatic tissues in rats. Daphnetin reduced pro-inflammatory cytokines and increased anti-inflammatory cytokines in rat SAP. Decreased expression of TLR4 and suppressed NF-κB signaling pathway.	*in vivo*	rat severe acute pancreatitis (SAP) model	(185)

### Coumarins and Hepatocellular Carcinoma

Over 900,000 new cases of hepatocellular carcinoma (HCC) are diagnosed annually worldwide, and it is the third main cause of cancer-associated death (186, 187). The stage of HCC at diagnosis is strongly associated with prognosis (187). Traditional chemotherapy agents for advanced-stage HCC are often ineffective. The development of new surgical approaches for HCC have improved the 5-year survival rate (188). If HCC is resistant to chemo-radiotherapy, it can spread to the lungs, brain, adrenal glands, and lymph nodes (188). Researchers are trying to understand more about the origin and progression of HCC, and the relevant molecular mechanisms (189–191), but more studies are needed to discover new agents for improved HCC treatment. Misfolded proteins and damaged organelles are destroyed within the cell by autophagy and lysosomal cleavage (192). Autophagy is essential for cell survival (193, 194). Many natural compounds have been found to have anti-cancer effects by promoting autophagy (195, 196). Cui et al. studied HepG2 and MHCC97 (two human HCC cell lines) treated with hydroxypyridinone-coumarin (HPC) *in vitro* (197). The MTT cytotoxicity test assessed proliferation and viability with and without HPC treatment. Cell autophagosomes were tagged with GFP-LC3 and visualized with confocal fluorescence microscopy, and Western blotting measured protein expression. HPC treatment reduced HepG2 cell proliferation by 29% and MHCC97 proliferation by 36%. HPC treatment increased expression of Atg-3, Atg5, LC3-II (LC3-phosphatidylethanolamine conjugate), and beclin-1, but decreased protein expression of Akt and p62. HPC treatment increased autophagy in MHCC97 and HepG2 cells as shown by GFP-LC3B fluorescence labeling. HPC also induced phosphorylation of ERK1/2 and led to Akt pathway down-regulation (197).

The hydrazide-hydrazone molecular structure is a new scaffold in medicinal chemistry that can inhibit both *α*- and *β*-glucosidase enzymes and potentially show anti-cancer activity (184, 198–206). The potential mechanisms of this are fourfold. Firstly, the hydrazide-hydrazone moiety is a metal chelator that can remove the catalytic zinc ion from metalloenzymes (207, 208); Secondly, it can form stable hydrogen bonds to its targets. Thirdly, its molecular structure includes an amino group, which can serve as a stand-in for aza-substituted amino acids. Fourthly the keto-enol tautomerization (*E*- and *Z*-forms) may orient and fix the conjugated hydrophobic substituent into the protein binding pocket *via* the best fit configuration (209). The formation of molecular hybrids between coumarins and hydrazide-hydrazone moieties might lead to novel anti-cancer compounds.

Nasr and colleagues evaluated a new series of coumarin hydrazide-hydrazone derivatives, for their activity against leukemia (CCRF), resistant pancreatic carcinoma (Panc-1), and hepatocellular carcinoma (HepG2) cancer cells *in vitro* (175). One of the novel coumarin hydrazide-hydrazone hybrids, bromocoumarin hydrazide-hydrazone derivative (BCHHD) 11b, demonstrated outstanding anti-cancer activity against cancer cells. Furthermore, BCHHD 11b triggered apoptosis by activating caspases 3/7, and led to inhibition of cell metabolism. Inhibition of CYP3A4 and GST led to cell death in a 11b dose-dependent manner. Furthermore, microarray evaluation revealed up-regulation and down-regulation of genes associated with tumor growth, cell cycle, and apoptosis. Moreover, BCHHD 11b could be not only useful in chemotherapy, but also could be used as a transporter for ^99m^Tc *in vivo*, as a radiopharmaceutical imaging agent for cancer treatment (170, 175).

**Table 4** lists some studies on anti-HCC cancer effects of coumarins.

**Table 4:** Anti-hepatocellular cancer effects of coumarins.

Coumarin compound	Dose	Mechanisms	Model	Cell line	Ref
Hydroxypyridinone-coumarin	2 µM	Induced autophagy, inhibited proliferation, activated ERK1/2, down-regulated the Akt pathway	*In vitro*	MHCC97 HepG	(210)
Furanocoumarin	100 μM	Anti cancer effect	*In vitro*	HepG2	(134)
Coumarin-3-carboxylic acid	0–1000 µM.	Inhibited DNA synthesis not by intercalation. Ames tests showed that all the tested agents or phase I metabolites were non-mutagenic	*In vitro*	CHANG Hep-G2	(211)
Esculetin	2.24 mM	Triggered mitochondrial caspase-dependent apoptosis	*In vivo* *In vitro*	Hepa1-6	(212)
Osthole	161.4 mM 137.0 mM	Inhibited HCC growth *in vivo* and *in vitro*, induced apoptosis by repressing NF-kB, increased expression of apoptosis-related genes.	*In vivo*	Hepa1-6 HepG2	(213)
4-Hydroxy-3-nitro-coumarin Ligand silver + 4-oxy-3-nitro-coumarin-bis (phenanthroline)	0, 20, 40, 80 μM for 4 h or 24 h IC50 at 4h = 80 µM and at 24 h =40 μM	Inhibited proliferation	*In vitro*	HepG2 CHANG	(214)
Coumarin-dioxy-acetic acid (cdoa) copper-coumarin-dioxyacetic acetate-phenathroline [Cu(cdoa)(phen)2]	0, 7.5, 15, 30 µM	[Cu(cdoa)(phen)2] inhibited proliferation more than the parent ligand [CdoaH2], phen, or the simple salt. Biochemical and morphological features consistent with both necrotic and apoptotic cell death	*In vitro*	Hep-G2	(215)
**Novel synthetic coumarins**	50 µM	Inhibited expression of NF-kB targeted genes	*In vitro*	HepG2	(216)
Natural coumarin	4.9 µM	Increased necrosis Inhibited tumor growth	*In vitro* *In vivo*	HepG2	(217)
Clausarin, dentatin, nordentatin, xantoxyletin	IC50 (µM) 17.6 ± 2.1 47.6 ± 2.8 29.9 ± 3.2 78.2 ± 2.2	Clausarin had the highest selective cytotoxicity. Xantoxyletin caused apoptosis and lowest necrosis in HepG2 cells after 24 h	*In vitro*	HepG2	(218)
**Coumarin-triazole hybrid**	IC50 = 0.80 μM.	Inhibited proliferation	*In vitro*	HepG2	(219)
**Thiazolylpyrazolyl coumarin derivatives**	IC50 = 5.4 – 10.7 µM	Anticancer activity	*In vitro*	HepG2	(220)
**Coumarin hybrids**	IC50 = 0.49-3.96μM	Inhibited proliferation	*In vitro*	Hep G2	(221)
**7,8-Dihydroxy-3-(4-nitrophenyl) coumarin**	IC50 = 17.65 μM	Cell cycle arrest at S phase, loss of mitochondrial membrane potential, mediated ROS-independent cell death	*In vitro*	HepG2	(177)
7-Hydroxy-6,8-dimethoxy-2*H*-1-benzopyran-2-one (isofraxidin)	IC50 = 100 μM	Inhibited invasion without influencing proliferation or attachment. Inhibited TPA-induced matrix metalloproteinase-7 (MMP-7) at both protein and mRNA levels. More effective at low cell density than at high density. Inhibited phosphorylation of ERK1/2, without affecting NF-kB nuclear translocation, activator protein-1 (AP-1) DNA binding activity, or degradation of IkB.	*In vitro*	HuH-7, HepG2	(222)
Juglansoside C extracted from bark of *Juglans mandshurica*.	IC50 = 70.9 μM	Showed moderate cytotoxicity Induced apoptosis in Hep3B cells	*In vitro*	Hep3B	(223)
7-OH-4-Methylcoumarin	IC50 = 356 µM	Inhibited proliferation in a dose-dependent manner. Reversed malignant phenotype and caused re-differentiation.	*In vitro*	SMMC-7721	(224)

### Coumarin and Esophageal Cancer

The most prevalent type of esophageal cancer is esophageal squamous cell carcinoma (ESCC). The rate of ESCC incidence varies by country, but the highest rates are seen in regions within the esophageal cancer belt, which runs from North of Iran to North and Central China (225).. The early stages of ESCC show only vague symptoms leading to delayed diagnosis, and the presence of drug-resistant cells has a detrimental effect on the results of standard chemotherapy regimens (226, 227). Recently, there has been a focus on cancer stem cells (CSCs) as a target for more effective cancer therapy, because it has been found that CSCs are largely responsible for recurrence, therapy resistance, and metastasis in several human cancer types (228). Various molecular markers are used to identify CSCs in different human malignancies. For instance, CSCs are positive for CD15, CD44, CD90, CXCR4, and CD133 antigens in ESCC (229–231).

Auraptene (AUR), or 7-geranyloxycoumarin, is a naturally occurring prenyloxycoumarin found in plants within the *Apiaceae* and *Rutaceae* families. AUR has been found to have different biological benefits such as, antioxidant, anti-inflammatory, anti-protozoal, anti-fungal, antibacterial, and immune system boosting activities (232). AUR supplements have been tested in animal cancer models, and it was shown that they had chemopreventive effects in GI, liver, skin, prostate, and breast cancer (233–242). The mechanisms of AUR chemoprevention include inhibition of lipid peroxidation, induction of glutathione S transferase activity, regulation of inflammation, and inhibition of superoxide generation (233, 235, 236, 242). Furthermore, researchers have shown that AUR has anti-cancer effects *in vitro*, and can inhibit the proliferation of breast and renal cancer cell lines, and can induce apoptosis in gastric, colon cancer, and leukemia cells. Some studies have shown the anti-cancer activity of AUR against colon CSCs, and AUR could inhibit the recurrence of colon tumors (243–248).

Saboor-Maleki and his colleagues investigated the anti-cancer activity of AUR on ESCC cells and CSCs looking at specific markers (249). They used the KYSE30 ESCC cell line to examine the effects of AUR and combinations with 5-fluorouracil, cisplatin, and paclitaxel. Furthermore, they used qRT-PCR to evaluate the expression of p21 and p53 (two tumor suppressor genes), BMI-1 (B cell-specific Moloney murine leukemia virus integration site 1), and CD44 (cluster of differentiation 44). Their findings revealed that AUR increased the cytotoxicty of 5-fluorouracil, paclitaxel, and cisplatin in KYSE30 cells, and the maximal effect was seen after 72 hours of AUR treatment, which induced apoptosis. In addition, qRT-PCR revealed that p53 and p21 were up-regulated, but BMI-1 and CD44 were down-regulated after AUR treatment. AUR inhibited esophageal cancer stem-like cells by increasing the effects of chemotherapy, and down-regulating BMI-1 and CD44 (249).

Osthole (7-methoxy-8-(3-methyl-2-butenyl)-2H-1-benzopyran-2-one) has the chemical formula C15H16O3, and is a biologically active coumarin compound isolated from *Fructus cnidii*, a herb used in traditional Chinese medicine to manage rheumatic pain, lumbar pain, and impotence (250, 251). Earlier studies have shown that osthole has various medical properties, including vasodilation, anti-osteoporosis, anti-inflammation, anti-allergy, and anti-seizure activity (252–257). In addition, osthole can trigger cellular apoptosis and suppress proliferation and metastasis in tumor cells, and may inhibit tumorigenesis (258–260).

In a study by Zhu et al., the proliferation of ESCC cells was suppressed by osthole in a time – and dose-dependent manner (261). Additionally, osthole caused cell cycle arrest at G2/M phase and triggered apoptosis. Moreover, cleaved caspase 9, cleaved PARP1, cleaved caspase3, and BAX were up-regulated, while the expression level of survivin, cyclin B1, PARP1, Bcl-2, and Cdc2, were decreased Osthole increased PTEN expression and decreased p-AKT (phosphorylated AKT) and PI3K, thereby modulating the PTEN-PI3K/AKT signaling pathway. Consequently, osthole may have a role in managing patients suffering from ESCC (261).

## Conclusions

Coumarin compounds have a broad range of biological activity, and consequently for decades many scientists have investigated these compounds, and some have devised new related structures to potentially treat cancer, as well as a plethora of other diseases. Coumarins play a crucial role in numerous biological processes such as antioxidant systems, regulation of cell growth, and chemoprevention from various disorders. Coumarin compounds have anti-cancer activity by regulating cell differentiation, growth, and the immune system responses. Therefore, coumarins can be combined with conventional drugs, to produce novel antitumor treatments with higher efficacy and fewer adverse effects.

Various synthetic methods such as the Knoevenagel, Pechmann, Perkin, Wittig, and Claisen reactions have been used to prepare coumarins as well as a diverse range of derivatives. Thanks to theses new molecular manipulation techniques, analogs with more potent activity and a higher therapeutic index have been discovered, even though coumarin itself and some of its natural compounds may show hepatotoxicity, which may limit their clinical use. Recent studies have shown that the antitumor effects of coumarins may be increased by the addition of various substituents to specific areas of the coumarin structure. As a result, this approach has led to the identification of some novel antitumor compounds.

Moreover, both synthetic and natural coumarins have been found to modulate specific signaling pathways, providing mechanistic explanations for their antitumor activity. Coumarin and its derivatives have promising antitumor properties and may result in novel antitumor drug regimens, however further laboratory studies are required before large scale clinical trials can be undertaken.

## Author Contributions

HM involved to conception, design, statistical analysis and drafting of the manuscript. ZB, SMM, FD, MRM, KM, FA, MA, AR, MC, MT and MRH contributed to data collection and manuscript drafting. All authors contributed to the article and approved the submitted version.

## Conflict of Interest

The authors declare that the research was conducted in the absence of any commercial or financial relationships that could be construed as a potential conflict of interest.

## Publisher’s Note

All claims expressed in this article are solely those of the authors and do not necessarily represent those of their affiliated organizations, or those of the publisher, the editors and the reviewers. Any product that may be evaluated in this article, or claim that may be made by its manufacturer, is not guaranteed or endorsed by the publisher.

## References

[1] Jemal A Bray F Center MM Ferlay J Ward E Forman D. Global Cancer Statistics. CA Cancer J Clin (2011) 61(2):69–90. doi: 10.3322/caac.20107 21296855

[2] Al-Ishaq RK Overy AJ Büsselberg D. Phytochemicals and Gastrointestinal Cancer: Cellular Mechanisms and Effects to Change Cancer Progression. Biomolecules (2020) 10(1). doi: 10.3390/biom10010105 PMC7022462 31936288

[3] Derakhshan MH Yazdanbod A Sadjadi AR Shokoohi B McColl KE Malekzadeh R. High Incidence of Adenocarcinoma Arising From the Right Side of the Gastric Cardia in NW Iran. Gut (2004) 53(9):1262–6. doi: 10.1136/gut.2003.035857 PMC1774166 15306582

[4] Zali H Rezaei-Tavirani M Azodi M. Gastric Cancer: Prevention, Risk Factors and Treatment. Gastroenterol Hepatol Bed Bench (2011) 4(4):175–85. PMC4017429 24834180

[5] Sitarz R Skierucha M Mielko J Offerhaus GJA Maciejewski R Polkowski WP. Gastric Cancer: Epidemiology, Prevention, Classification, and Treatment. Cancer Manag Res (2018) 10:239–48. doi: 10.2147/CMAR.S149619 PMC5808709 29445300

[6] Holian O Wahid S Atten MJ Attar BM. Inhibition of Gastric Cancer Cell Proliferation by Resveratrol: Role of Nitric Oxide. Am J Physiol Gastrointest Liver Physiol (2002) 282(5):G809–16. doi: 10.1152/ajpgi.00193.2001 11960777

[7] Zhou XM Wong BC Fan XM Zhang HB Lin MC Kung HF. Non-Steroidal Anti-Inflammatory Drugs Induce Apoptosis in Gastric Cancer Cells Through Up-Regulation of Bax and Bak. Carcinogenesis (2001) 22(9):1393–7. doi: 10.1093/carcin/22.9.1393 11532860

[8] Hundahl SA Phillips JL Menck HR. The National Cancer Data Base Report on Poor Survival of U.S. Gastric Carcinoma Patients Treated With Gastrectomy: Fifth Edition American Joint Committee on Cancer Staging, Proximal Disease, and the "Different Disease" Hypothesis. Cancer (2000) 88(4):921–32. 10679663

[9] Correa P. Gastric Cancer: Overview. Gastroenterol Clin North Am (2013) 42(2):211–7. doi: 10.1016/j.gtc.2013.01.002 PMC3995345 23639637

[10] Jafarpour SM Safaei M Mohseni M Salimian M Aliasgharzadeh A Farhood B. The Radioprotective Effects of Curcumin and Trehalose Against Genetic Damage Caused By I-131. Indian J Nucl Med (2018) 33(2):99–104. 29643668 10.4103/ijnm.IJNM_158_17 PMC5883450

[11] Motallebzadeh E Tameh AA Zavareh SAT Farhood B Aliasgharzedeh A Mohseni M. Neuroprotective Effect of Melatonin on Radiation-Induced Oxidative Stress and Apoptosis in the Brainstem of Rats. J Cell Physiol (2020) 235(11):8791–8. doi: 10.1002/jcp.29722 32324264

[12] Pal D Saha S. Coumarins: An Important Phytochemical With Therapeutic Potential. In: Plant-Derived Bioactives. New York City, USA: Springer (2020). p. 205–22.

[13] Venugopala KN Rashmi V Odhav B. Review on Natural Coumarin Lead Compounds for Their Pharmacological Activity. BioMed Res Int (2013) 2013:963248. doi: 10.1155/2013/963248 23586066 PMC3622347

[14] Singh H Singh JV Bhagat K Gulati HK Sanduja M Kumar N. Rational Approaches, Design Strategies, Structure Activity Relationship and Mechanistic Insights for Therapeutic Coumarin Hybrids. Bioorg Med Chem (2019) 27(16):3477–510. doi: 10.1016/j.bmc.2019.06.033 PMC7970831 31255497

[15] Wu Y Xu J Liu Y Zeng Y Wu G. A Review on Anti-Tumor Mechanisms of Coumarins. Front Oncol (2020) 10:592853. doi: 10.3389/fonc.2020.592853 33344242 PMC7746827

[16] Lake BG. Coumarin Metabolism, Toxicity and Carcinogenicity: Relevance for Human Risk Assessment. Food Chem Toxicol (1999) 37(4):423–53. doi: 10.1016/S0278-6915(99)00010-1 10418958

[17] Cooke D. Studies on the Mode of Action of Coumarins (Coumarin, 6-Hydroxycoumarin, 7-Hydroxycoumarin & Esculetin) at a Cellular Level. Dublin, Ireland: Dublin City University (1999).

[18] Ritschel W KW G. Biopharmaceutical Parameters of Coumarin and 7-Hydroxycoumarin. Die Pharma. Ind. (1981) 43:271–6.

[19] Egan DA O’Kennedy R. Spectrofluorimetric Method for the Quantification of 7-Hydroxycoumarin in Urine and Plasma Using Both Extracted and Unextracted Samples. Analyst (1993) 118(2):201–3. doi: 10.1039/an9931800201 8442515

[20] Dempsey E O'Sullivan C Smyth MR Egan D O'Kennedy R Wang J. Development of an Antibody-Based Amperometric Biosensor to Study the Reaction of 7-Hydroxycoumarin With its Specific Antibody. Analyst (1993) 118(4):411–3. doi: 10.1039/an9931800411 8494174

[21] Keating GJ. Biosensor-Based Studies on Coumarins. Dublin, Ireland: Dublin City University (1998).

[22] Pelkonen O Rautio A Raunio H Pasanen M. CYP2A6: A Human Coumarin 7-Hydroxylase. Toxicology (2000) 144(1-3):139–47. doi: 10.1016/S0300-483X(99)00200-0 10781881

[23] Pelkonen O Mäenpää J Taavitsainen P Rautio A Raunio H. Inhibition and Induction of Human Cytochrome P450 (CYP) Enzymes. Xenobiotica (1998) 28(12):1203–53. doi: 10.1080/004982598238886 9890159

[24] Pelkonen O Raunio H Rautio A Pasanen M Lang M. The Metabolism of Coumarins. In: Coumarins: Biology, Applications and Mode of Action. Wiley (1997).

[25] Taavitsainen P. Cytochrome P450 Isoform-Specific In Vitro Methods to Predict Drug Metabolism and Interactions. Dissertation. Pentti Kaiteran katu, Oulu, Finland: Faculty of Medicine, University of Oulu (2001).

[26] Bogan DP Deasy B O'Kennedy R Smyth MR Fuhr U. Determination of Free and Total 7-Hydroxycoumarin in Urine and Serum by Capillary Electrophoresis. J Chromatogr B BioMed Appl (1995) 663(2):371–8. doi: 10.1016/0378-4347(94)00444-A 7735485

[27] Rautio A Kraul H Kojo A Salmela E Pelkonen O. Interindividual Variability of Coumarin 7-Hydroxylation in Healthy Volunteers. Pharmacogenetics (1992) 2(5):227–33. doi: 10.1097/00008571-199210000-00005 1306122

[28] Egan D O’Kennedy R. The Production and Characterisation of Anti-7-Hydroxycoumarin Antibodies and Their Use in the Development of an Enzyme-Linked Immunosorbent Assay. J Ir Coll Physicians Surg (1993) 22:72.

[29] Bogan DP Deasy B O'Kennedy R Smyth MR. Interspecies Differences in Coumarin Metabolism in Liver Microsomes Examined by Capillary Electrophoresis. Xenobiotica (1996) 26(4):437–45. doi: 10.3109/00498259609046722 9173684

[30] Pineo GF Hull RD. Adverse Effects of Coumarin Anticoagulants. Drug Saf (1993) 9(4):263–71. doi: 10.2165/00002018-199309040-00004 8260120

[31] Aronson JK. Meyler’s Side Effects of Drugs: The International Encyclopedia of Adverse Drug Reactions and Interactions. Amsterdam, Netherland: Elsevier (2015).

[32] Hart JP Catterall A Dodds RA Klenerman L Shearer MJ Bitensky L. Circulating Vitamin K1 Levels in Fractured Neck of Femur. Lancet (1984) 2(8397):283. doi: 10.1016/S0140-6736(84)90321-0 6146829

[33] Ginsberg JS Hirsh J. Use of Antithrombotic Agents During Pregnancy. Chest (1992) 102(4 Suppl):385s–90s. doi: 10.1378/chest.102.4_Supplement.385S 1395823

[34] Murray RDH Méndez J Brown SA. The Natural Coumarins. New Jersey, United States: Weliy.Hoboken (1982).

[35] Ojala T. Biological Screening of Plant Coumarins. Richmond, United Kingdom (2001).

[36] Keating G O’kennedy R. The Chemistry and Occurrence of Coumarins. In: Coumarins: Biology, Applications and Mode of Action. New Jersey, United States: Weliy.Hoboken. (1997) p. 23–66.

[37] Trease GE Evans WC. Pharmacognosy. (1983).

[38] Ahmad J Shamsuddin K Zaman A. Pyranocoumarin From Atalantia Ceylanica. Phytochemistry (1984) 28(9):2098–9. doi: 10.1016/S0031-9422(00)84993-6

[39] Lacy A O’Kennedy R. Studies on Coumarins and Coumarin-Related Compounds to Determine Their Therapeutic Role in the Treatment of Cancer. Curr Pharm Des (2004) 10(30):3797–811. doi: 10.2174/1381612043382693 15579072

[40] Kresge N Simoni RD Hill RL. Hemorrhagic Sweet Clover Disease, Dicumarol, and Warfarin: The Work of Karl Paul Link. J Biol Chem (2005) 280(8):e5–5.

[41] Pan L Chai H Kinghorn AD. The Continuing Search for Antitumor Agents From Higher Plants. Phytochem Lett (2010) 3(1):1–8. doi: 10.1016/j.phytol.2009.11.005 20228943 PMC2836022

[42] Majnooni MB Fakhri S Shokoohinia Y Mojarrab M Kazemi-Afrakoti S Farzaei MH. Isofraxidin: Synthesis, Biosynthesis, Isolation, Pharmacokinetic and Pharmacological Properties. Molecules (2020) 25(9). doi: 10.3390/molecules25092040 PMC7248759 32349420

[43] Pádua D Rocha E Gargiulo D Ramos A. Bioactive Compounds From Brown Seaweeds: Phloroglucinol, Fucoxanthin and Fucoidan as Promising Therapeutic Agents Against Breast Cancer. Phytochem Lett (2015) 14:91–8. doi: 10.1016/j.phytol.2015.09.007

[44] Bourgaud F Hehn A Larbat R Doerper S Gontier E Kellner S. Biosynthesis of Coumarins in Plants: A Major Pathway Still to be Unravelled for Cytochrome P450 Enzymes. Phytochem Rev (2006) 5(2-3):293–308. doi: 10.1007/s11101-006-9040-2

[45] Baghdadi MA Al-Abbasi FA El-Halawany AM Aseeri AH Al-Abd AM. Anticancer Profiling for Coumarins and Related O-Naphthoquinones From Mansonia Gagei Against Solid Tumor Cells *In Vitro* . Molecules (2018) 23(5). doi: 10.3390/molecules23051020 PMC6102575 29701706

[46] Neri D Supuran CT. Interfering With pH Regulation in Tumours as a Therapeutic Strategy. Nat Rev Drug Discov (2011) 10(10):767–77. doi: 10.1038/nrd3554 21921921

[47] Supuran CT. Carbonic Anhydrase Inhibition and the Management of Hypoxic Tumors. Metabolites (2017) 7(3). doi: 10.3390/metabo7030048 PMC5618333 28926956

[48] Zengin Kurt B Sonmez F Ozturk D Akdemir A Angeli A Supuran CT. Synthesis of Coumarin-Sulfonamide Derivatives and Determination of Their Cytotoxicity, Carbonic Anhydrase Inhibitory and Molecular Docking Studies. Eur J Med Chem (2019) 183:111702. doi: 10.1016/j.ejmech.2019.111702 31542715

[49] Kumar S. Caspase Function in Programmed Cell Death. Cell Death Differ (2007) 14(1):32–43. doi: 10.1038/sj.cdd.4402060 17082813

[50] Nordin N Pulchrin A. A New Natural Coumarin Derivative of Enicosanthellum Pulchrum, Induces Apoptosis in Ovarian Cancer Cells *via* Intrinsic Pathway. PloS One (2016) 11(5):e0154023. doi: 10.1371/journal.pone.0154023 27136097 PMC4852948

[51] Yu JS Cui W. Proliferation, Survival and Metabolism: The Role of PI3K/AKT/mTOR Signalling in Pluripotency and Cell Fate Determination. Development (2016) 143(17):3050–60. doi: 10.1242/dev.137075 27578176

[52] Wee P Wang Z. Epidermal Growth Factor Receptor Cell Proliferation Signaling Pathways. Cancers (Basel) (2017) 9(5). doi: 10.3390/cancers9050052 PMC5447962 28513565

[53] Martini M De Santis MC Braccini L Gulluni F Hirsch E. PI3K/AKT Signaling Pathway and Cancer: An Updated Review. Ann Med (2014) 46(6):372–83. doi: 10.3109/07853890.2014.912836 24897931

[54] Mayer IA Arteaga CL. The PI3K/AKT Pathway as a Target for Cancer Treatment. Annu Rev Med (2016) 67:11–28. doi: 10.1146/annurev-med-062913-051343 26473415

[55] Guo H He Y Bu C Peng Z. Antitumor and Apoptotic Effects of 5-Methoxypsoralen in U87MG Human Glioma Cells and its Effect on Cell Cycle, Autophagy and PI3K/Akt Signaling Pathway. Arch Med Sci (2019) 15(6):1530–8. doi: 10.5114/aoms.2019.81729 PMC6855158 31749882

[56] Yao D Pan D Zhen Y Huang J Wang J Zhang J. Ferulin C Triggers Potent PAK1 and P21-Mediated Anti-Tumor Effects in Breast Cancer by Inhibiting Tubulin Polymerization *In Vitro* and *In Vivo* . Pharmacol Res (2020) 152:104605. doi: 10.1016/j.phrs.2019.104605 31863866

[57] Bray F Ferlay J Soerjomataram I Siegel RL Torre LA Jemal A. Global Cancer Statistics 2018: GLOBOCAN Estimates of Incidence and Mortality Worldwide for 36 Cancers in 185 Countries. CA Cancer J Clin (2018) 68(6):394–424. doi: 10.3322/caac.21492 30207593

[58] Cruz-Monteagudo M González-Díaz H. Unified Drug-Target Interaction Thermodynamic Markov Model Using Stochastic Entropies to Predict Multiple Drugs Side Effects. Eur J Med Chem (2005) 40(10):1030–41. doi: 10.1016/j.ejmech.2005.04.012 15951063

[59] Kerru N Singh P Koorbanally N Raj R Kumar V. Recent Advances (2015-2016) in Anticancer Hybrids. Eur J Med Chem (2017) 142:179–212. doi: 10.1016/j.ejmech.2017.07.033 28760313

[60] Pałasz A Cież D. Search of Uracil Derivatives as Bioactive Agents. Uracils and Fused Uracils: Synthesis, Biological Activity and Applications. Eur J Med Chem (2015) 97:582–611. doi: 10.1016/j.ejmech.2014.10.008 25306174

[61] Legraverend M Grierson DS. The Purines: Potent and Versatile Small Molecule Inhibitors and Modulators of Key Biological Targets. Bioorg Med Chem (2006) 14(12):3987–4006. doi: 10.1016/j.bmc.2005.12.060 16503144

[62] Galmarini CM Mackey JR Dumontet C. Nucleoside Analogues and Nucleobases in Cancer Treatment. Lancet Oncol (2002) 3(7):415–24. doi: 10.1016/S1470-2045(02)00788-X 12142171

[63] Parker WB. Enzymology of Purine and Pyrimidine Antimetabolites Used in the Treatment of Cancer. Chem Rev (2009) 109(7):2880–93. doi: 10.1021/cr900028p PMC2827868 19476376

[64] Calderón-Arancibia J Espinosa-Bustos C Cañete-Molina Á Tapia RA Faúndez M Torres MJ. Synthesis and Pharmacophore Modelling of 2,6,9-Trisubstituted Purine Derivatives and Their Potential Role as Apoptosis-Inducing Agents in Cancer Cell Lines. Molecules (2015) 20(4):6808–26. doi: 10.3390/molecules20046808 PMC6272238 25884555

[65] Kolb HC Sharpless KB. The Growing Impact of Click Chemistry on Drug Discovery. Drug Discov Today (2003) 8(24):1128–37. doi: 10.1016/S1359-6446(03)02933-7 14678739

[66] Krištafor S Bistrović A Plavec J Makuc D Martinović T Pavelić SK. One-Pot Click Synthesis of 1, 2, 3-Triazole-Embedded Unsaturated Uracil Derivatives and Hybrids of 1, 5-and 2, 5-Disubstituted Tetrazoles and Pyrimidines. Tetrahedron Lett (2015) 56(10):1222–8. doi: 10.1016/j.tetlet.2015.01.152

[67] Vijaya Raghava Reddy L Venkat Reddy P Mishra NN Shukla PK Yadav G Srivastava R. Synthesis and Biological Evaluation of Glycal-Derived Novel Tetrahydrofuran 1,2,3-Triazoles by ’Click’ Chemistry. Carbohydr Res (2010) 345(11):1515–21. doi: 10.1016/j.carres.2010.03.031 20478557

[68] Gupta A Kumar BS Negi AS. Current Status on Development of Steroids as Anticancer Agents. J Steroid Biochem Mol Biol (2013) 137:242–70. doi: 10.1016/j.jsbmb.2013.05.011 23727548

[69] Hu H Rao Z Feng M Wu Z Xu J Chen H. 3,16-Bisquaternary Ammonium Steroid Derivatives as Neuromuscular Blocking Agents: Synthesis and Biological Evaluation. Steroids (2015) 96:103–14. doi: 10.1016/j.steroids.2015.01.008 25637675

[70] Huang LH Zheng YF Song CJ Wang YG Xie ZY Lai YW. Synthesis of Novel D-Ring Fused 7’-Aryl-Androstano[17,16-D][1,2,4] Triazolo[1,5-a]Pyrimidines. Steroids (2012) 77(5):367–74. doi: 10.1016/j.steroids.2011.12.012 22182831

[71] Huang LH Zheng YF Lu YZ Song CJ Wang YG Yu B. Synthesis and Biological Evaluation of Novel Steroidal[17,16-D][1,2,4]Triazolo[1,5-a]Pyrimidines. Steroids (2012) 77(6):710–5. doi: 10.1016/j.steroids.2012.03.002 22445685

[72] Yu B Shi XJ Qi PP Yu DQ Liu HM. Design, Synthesis and Biological Evaluation of Novel Steroidal Spiro-Oxindoles as Potent Antiproliferative Agents. J Steroid Biochem Mol Biol (2014) 141:121–34. doi: 10.1016/j.jsbmb.2014.01.015 24508598

[73] Guo H Zhang G Zhang T He X Wu Z Xiao Y. Synthesis, Characterization and Biological Evaluation of Some 16β-Azolyl-3β-Amino-5α-Androstane Derivatives as Potential Anticancer Agents. Eur J Med Chem (2011) 46(9):3662–74. doi: 10.1016/j.ejmech.2011.05.030 21641694

[74] Zhao JW Wu ZH Guo JW Huang MJ You YZ Liu HM. Synthesis and Anti-Gastric Cancer Activity Evaluation of Novel Triazole Nucleobase Analogues Containing Steroidal/Coumarin/Quinoline Moieties. Eur J Med Chem (2019) 181:111520. doi: 10.1016/j.ejmech.2019.07.023 31404863

[75] Wei Y Ito Y. Preparative Isolation of Imperatorin, Oxypeucedanin and Isoimperatorin From Traditional Chinese Herb “Bai Zhi”Angelica Dahurica (Fisch. Ex Hoffm) Benth. Et Hook Using Multidimensional High-Speed Counter-Current Chromatography. J Chromatogr A (2006) 1115(1-2):112–7. 10.1016/j.chroma.2006.02.081 16542667

[76] Kim YK Kim YS Ryu SY. Antiproliferative Effect of Furanocoumarins From the Root of Angelica Dahurica on Cultured Human Tumor Cell Lines. Phytother Res (2007) 21(3):288–90. doi: 10.1002/ptr.2043 17143927

[77] Moon TC Jin M Son JK Chang HW. The Effects of Isoimperatorin Isolated From Angelicae Dahuricae on Cyclooxygenase-2 and 5-Lipoxygenase in Mouse Bone Marrow-Derived Mast Cells. Arch Pharm Res (2008) 31(2):210–5. doi: 10.1007/s12272-001-1143-0 18365692

[78] Kim DK Lim JP Yang JH Eom DO Eun JS Leem KH. Acetylcholinesterase Inhibitors From the Roots of Angelica Dahurica. Arch Pharm Res (2002) 25(6):856–9. doi: 10.1007/BF02977004 12510838

[79] Park HY Kwon SB Heo NK Chun WJ Kim MJ Kwon YS. Constituents of the Stem of Angelica Gigas With Rat Lens Aldose Reductase Inhibitory Activity. J Korean Soc Appl Biol Chem (2011) 54(2):194–9. doi: 10.3839/jksabc.2011.032

[80] Baek NI Ahn EM Kim HY Park YD. Furanocoumarins From the Root of Angelica Dahurica. Arch Pharm Res (2000) 23(5):467–70. doi: 10.1007/BF02976574 11059825

[81] Kleiner HE Reed MJ DiGiovanni J. Naturally Occurring Coumarins Inhibit Human Cytochromes P450 and Block Benzo[a]Pyrene and 7,12-Dimethylbenz[a]Anthracene DNA Adduct Formation in MCF-7 Cells. Chem Res Toxicol (2003) 16(3):415–22. doi: 10.1021/tx025636d 12641443

[82] Tong K Xin C Chen W. Isoimperatorin Induces Apoptosis of the SGC-7901 Human Gastric Cancer Cell Line *via* the Mitochondria-Mediated Pathway. Oncol Lett (2017) 13(1):518–24. doi: 10.3892/ol.2016.5387 PMC5244867 28123591

[83] Yang HB Gao HR Ren YJ Fang FX Tian HT Gao ZJ. Effects of Isoimperatorin on Proliferation and Apoptosis of Human Gastric Carcinoma Cells. Oncol Lett (2018) 15(5):7993–8. doi: 10.3892/ol.2018.8303 PMC5920862 29731910

[84] Perumalsamy H Sankarapandian K Veerappan K Natarajan S Kandaswamy N Thangavelu L. *In Silico* and *In Vitro* Analysis of Coumarin Derivative Induced Anticancer Effects by Undergoing Intrinsic Pathway Mediated Apoptosis in Human Stomach Cancer. Phytomedicine (2018) 46:119–30. doi: 10.1016/j.phymed.2018.04.021 30097112

[85] Shahverdi AR Fakhimi A Zarrini G Dehghan G Iranshahi M. Galbanic Acid From Ferula Szowitsiana Enhanced the Antibacterial Activity of Penicillin G and Cephalexin Against Staphylococcus Aureus. Biol Pharm Bull (2007) 30(9):1805–7. doi: 10.1248/bpb.30.1805 17827745

[86] Pimenov MG Leonov MVE. The Genera of the Umbelliferae: A Nomenclator. Richmond, United Kingdom: Royal Botanic Gardens, Kew (1993).

[87] Zhou P Takaishi Y Duan H Chen B Honda G Itoh M. Coumarins and Bicoumarin From Ferula Sumbul: Anti-HIV Activity and Inhibition of Cytokine Release. Phytochemistry (2000) 53(6):689–97. doi: 10.1016/S0031-9422(99)00554-3 10746882

[88] Ryu S-Y Lee C-O Choi S-u Park S-h Kim Y-S Kim S-k. Anticancer Composition Comprising Sesquiterpenes Isolated From Resina Ferulae. (2004), Google Patents.

[89] Mashinchian O Salehi R Dehghan G Aganejad A Davaran S Omidi Y. Novel Thermosensitive Poly (N-Isopropylacrylamide-Co-Vinylpyrrolidone-Co-Methacrylic Acid) Nanosystems for Delivery of Natural Products. Int J Drug Deliv (2010) 2(4). doi: 10.5138/ijdd.2010.0975.0215.02039

[90] Sarbolouki MN Sadeghizadeh M Yaghoobi MM Karami A Lohrasbi T. Dendrosomes: A Novel Family of Vehicles for Transfection and Therapy. J Chem Technol Biotechnol: Int Res Process Environ Clean Technol (2000) 75(10):919–22. doi: 10.1002/1097-4660(200010)75:10<919::AID-JCTB308>3.0.CO;2-S

[91] Sadeghizadeh M Ranjbar B Damaghi M Khaki L Sarbolouki MN Najafi F. Dendrosomes as Novel Gene Porters-III. J Chem Technol Biotechnol (2008) 83(6):912–20. doi: 10.1002/jctb.1891

[92] Babaei E Sadeghizadeh M Hassan ZM Feizi MA Najafi F Hashemi SM. Dendrosomal Curcumin Significantly Suppresses Cancer Cell Proliferation *In Vitro* and *In Vivo* . Int Immunopharmacol (2012) 12(1):226–34. doi: 10.1016/j.intimp.2011.11.015 22155627

[93] Dehghan Esmatabadi MJ Farhangi B Safari Z Kazerooni H Shirzad H Zolghadr F. Dendrosomal Curcumin Inhibits Metastatic Potential of Human SW480 Colon Cancer Cells Through Down-Regulation of Claudin1, Zeb1 and Hef1-1 Gene Expression. Asian Pac J Cancer Prev (2015) 16(6):2473–81. doi: 10.7314/APJCP.2015.16.6.2473 25824783

[94] Aas Z Babaei E Hosseinpour Feizi MA Dehghan G. Anti-Proliferative and Apoptotic Effects of Dendrosomal Farnesiferol C on Gastric Cancer Cells. Asian Pac J Cancer Prev (2015) 16(13):5325–9. doi: 10.7314/APJCP.2015.16.13.5325 26225673

[95] Li G Wang J Li X Xu J Zhang Z Si J. A New Terpene Coumarin Microbial Transformed by Mucor Polymorphosporus Induces Apoptosis of Human Gastric Cancer Cell Line MGC-803. Arch Pharm Res (2018) 41(6):646–54. doi: 10.1007/s12272-018-1028-0 PMC6028838 29619675

[96] Wang G Lu M Yao Y Wang J Li J. Esculetin Exerts Antitumor Effect on Human Gastric Cancer Cells Through IGF-1/PI3K/Akt Signaling Pathway. Eur J Pharmacol (2017) 814:207–15. doi: 10.1016/j.ejphar.2017.08.025 28847482

[97] Zhao JQ Wang YM Wang S Dang J Shi YP Mei LJ. A New Isocoumarin From the Aerial Parts of Aconitum Gymnandrum. Nat Prod Res (2016) 30(15):1746–52. doi: 10.1080/14786419.2015.1137574 26916990

[98] Pan H Wang BH Lv W Jiang Y He L. Esculetin Induces Apoptosis in Human Gastric Cancer Cells Through a Cyclophilin D-Mediated Mitochondrial Permeability Transition Pore Associated With ROS. Chem Biol Interact (2015) 242:51–60. doi: 10.1016/j.cbi.2015.09.015 26388407

[99] Mohareb RM MegallyAbdo NY. Uses of 3-(2-Bromoacetyl)-2H-Chromen-2-One in the Synthesis of Heterocyclic Compounds Incorporating Coumarin: Synthesis, Characterization and Cytotoxicity. Molecules (2015) 20(6):11535–53. doi: 10.3390/molecules200611535 PMC6272230 26111181

[100] Kim NH Kim SN Oh JS Lee S Kim YK. Anti-Mitotic Potential of 7-Diethylamino-3(2’-Benzoxazolyl)-Coumarin in 5-Fluorouracil-Resistant Human Gastric Cancer Cell Line SNU620/5-FU. Biochem Biophys Res Commun (2012) 418(4):616–21. doi: 10.1016/j.bbrc.2012.01.049 22301191

[101] Liu XH Liu HF Chen J Yang Y Song BA Bai LS. Synthesis and Molecular Docking Study of Novel Coumarin Derivatives Containing 4,5-Dihydropyrazole Moiety as Potential Antitumor Agents. Bioorg Med Chem Lett (2010) 20(19):5705–8. doi: 10.1016/j.bmcl.2010.08.017 20800480

[102] Rasul A Khan M Yu B Ma T Yang H. Xanthoxyletin, a Coumarin Induces S Phase Arrest and Apoptosis in Human Gastric Adenocarcinoma SGC-7901 Cells. Asian Pac J Cancer Prev (2011) 12(5):1219–23. 21875271

[103] Kawaii S Tomono Y Ogawa K Sugiura M Yano M Yoshizawa Y. The Antiproliferative Effect of Coumarins on Several Cancer Cell Lines. Anticancer Res (2001) 21(2a):917–23. 11396185

[104] van Lieshout EM Ekkel MP Bedaf MM Nijhoff WA Peters WH. Effects of Dietary Anticarcinogens on Rat Gastrointestinal Glutathione Peroxidase Activity. Oncol Rep (1998) 5(4):959–63. doi: 10.3892/or.5.4.959 9625855

[105] Weber US Steffen B Siegers CP. Antitumor-Activities of Coumarin, 7-Hydroxy-Coumarin and its Glucuronide in Several Human Tumor Cell Lines. Res Commun Mol Pathol Pharmacol (1998) 99(2):193–206. 9583093

[106] Siegel RL Miller KD Jemal A. Cancer Statistics, 2018. CA Cancer J Clin (2018) 68(1):7–30. doi: 10.3322/caac.21442 29313949

[107] Favoriti P Carbone G Greco M Pirozzi F Pirozzi RE Corcione F. Worldwide Burden of Colorectal Cancer: A Review. Updates Surg (2016) 68(1):7–11. doi: 10.1007/s13304-016-0359-y 27067591

[108] Fusco A Fedele M. Roles of HMGA Proteins in Cancer. Nat Rev Cancer (2007) 7(12):899–910. doi: 10.1038/nrc2271 18004397

[109] Shlapobersky M Sanders R Clark C Spector DH. Repression of HMGA2 Gene Expression by Human Cytomegalovirus Involves the IE2 86-Kilodalton Protein and is Necessary for Efficient Viral Replication and Inhibition of Cyclin A Transcription. J Virol (2006) 80(20):9951–61. doi: 10.1128/JVI.01300-06 PMC1617307 17005673

[110] Fedele M Visone R De Martino I Troncone G Palmieri D Battista S. HMGA2 Induces Pituitary Tumorigenesis by Enhancing E2F1 Activity. Cancer Cell (2006) 9(6):459–71. doi: 10.1016/j.ccr.2006.04.024 16766265

[111] Brabletz T Kalluri R Nieto MA Weinberg RA. EMT in Cancer. Nat Rev Cancer (2018) 18(2):128–34. doi: 10.1038/nrc.2017.118 29326430

[112] Huber MA Kraut N Beug H. Molecular Requirements for Epithelial-Mesenchymal Transition During Tumor Progression. Curr Opin Cell Biol (2005) 17(5):548–58. doi: 10.1016/j.ceb.2005.08.001 16098727

[113] Wang X Liu X Li AY Chen L Lai L Lin HH. Overexpression of HMGA2 Promotes Metastasis and Impacts Survival of Colorectal Cancers. Clin Cancer Res (2011) 17(8):2570–80. doi: 10.1158/1078-0432.CCR-10-2542 PMC3079060 21252160

[114] Klaunig JE. Oxidative Stress and Cancer. Curr Pharm Des (2018) 24(40):4771–8. 10.2174/1381612825666190215121712 30767733

[115] Menegon S Columbano A Giordano S. The Dual Roles of NRF2 in Cancer. Trends Mol Med (2016) 22(7):578–93. doi: 10.1016/j.molmed.2016.05.002 27263465

[116] Sadeghi MR Jeddi F Soozangar N Somi MH Samadi N. The Role of Nrf2-Keap1 Axis in Colorectal Cancer, Progression, and Chemoresistance. Tumour Biol (2017) 39(6):1010428317705510. doi: 10.1177/1010428317705510 28621229

[117] Saeidnia S Abdollahi M. Antioxidants: Friends or Foe in Prevention or Treatment of Cancer: The Debate of the Century. Toxicol Appl Pharmacol (2013) 271(1):49–63. doi: 10.1016/j.taap.2013.05.004 23680455

[118] Smith S. Telomerase Can’t Handle the Stress. Genes Dev (2018) 32(9-10):597–9. doi: 10.1101/gad.316042.118 PMC6004074 29802121

[119] Desideri E Ciccarone F Ciriolo MR. Targeting Glutathione Metabolism: Partner in Crime in Anticancer Therapy. Nutrients (2019) 11(8). doi: 10.3390/nu11081926 PMC6724225 31426306

[120] Peng X Gandhi V. ROS-Activated Anticancer Prodrugs: A New Strategy for Tumor-Specific Damage. Ther Deliv (2012) 3(7):823–33. doi: 10.4155/tde.12.61 PMC3566582 22900465

[121] Chen CH Hsieh YC Yang PM Liu YR Cho EC. Dicoumarol Suppresses HMGA2-Mediated Oncogenic Capacities and Inhibits Cell Proliferation by Inducing Apoptosis in Colon Cancer. Biochem Biophys Res Commun (2020) 524(4):1003–9. doi: 10.1016/j.bbrc.2020.01.147 32063361

[122] Aday B Ulus R Tanç M Kaya M Supuran CT. Synthesis of Novel 5-Amino-1,3,4-Thiadiazole-2-Sulfonamide Containing Acridine Sulfonamide/Carboxamide Compounds and Investigation of Their Inhibition Effects on Human Carbonic Anhydrase I, II, IV and VII. Bioorg Chem (2018) 77:101–5. doi: 10.1016/j.bioorg.2017.12.035 29353727

[123] Mohamed MA Abdel-Aziz AA Sakr HM El-Azab AS Bua S Supuran CT. Synthesis and Human/Bacterial Carbonic Anhydrase Inhibition With a Series of Sulfonamides Incorporating Phthalimido Moieties. Bioorg Med Chem (2017) 25(8):2524–9. doi: 10.1016/j.bmc.2017.03.017 28318894

[124] Gulçin İ Taslimi P. Sulfonamide Inhibitors: A Patent Review 2013-Present. Expert Opin Ther Pat (2018) 28(7):541–9. 10.1080/13543776.2018.1487400 29886770

[125] Gul HI Yamali C Yesilyurt F Sakagami H Kucukoglu K Gulcin I. Microwave-Assisted Synthesis and Bioevaluation of New Sulfonamides. J Enzyme Inhib Med Chem (2017) 32(1):369–74. doi: 10.1080/14756366.2016.1254207 PMC6009867 28260401

[126] Köksal Z Kalin R Camadan Y Usanmaz H Almaz Z Gülçin İ. Secondary Sulfonamides as Effective Lactoperoxidase Inhibitors. Molecules (2017) 22(6). doi: 10.3390/molecules22060793 PMC6152724 28538675

[127] Köksal Z Alım Z Bayrak S Gülçin İ Özdemir H. Investigation of the Effects of Some Sulfonamides on Acetylcholinesterase and Carbonic Anhydrase Enzymes. J Biochem Mol Toxicol (2019) 33(5):e22300. 30811749 10.1002/jbt.22300

[128] Nocentini A Carta F Ceruso M Bartolucci G Supuran CT. Click-Tailed Coumarins With Potent and Selective Inhibitory Action Against the Tumor-Associated Carbonic Anhydrases IX and XII. Bioorg Med Chem (2015) 23(21):6955–66. doi: 10.1016/j.bmc.2015.09.041 26432607

[129] De Luca L Mancuso F Ferro S Buemi MR Angeli A Del Prete S. Inhibitory Effects and Structural Insights for a Novel Series of Coumarin-Based Compounds That Selectively Target Human CA IX and CA XII Carbonic Anhydrases. Eur J Med Chem (2018) 143:276–82. doi: 10.1016/j.ejmech.2017.11.061 29197732

[130] Bozdag M Alafeefy AM Altamimi AM Vullo D Carta F Supuran CT. Coumarins and Other Fused Bicyclic Heterocycles With Selective Tumor-Associated Carbonic Anhydrase Isoforms Inhibitory Activity. Bioorg Med Chem (2017) 25(2):677–83. doi: 10.1016/j.bmc.2016.11.039 27939347

[131] Kurt BZ Dag A Doğan B Durdagi S Angeli A Nocentini A. Synthesis, Biological Activity and Multiscale Molecular Modeling Studies of Bis-Coumarins as Selective Carbonic Anhydrase IX and XII Inhibitors With Effective Cytotoxicity Against Hepatocellular Carcinoma. Bioorg Chem (2019) 87:838–50. doi: 10.1016/j.bioorg.2019.03.003 31003041

[132] Zengin Kurt B Sonmez F Durdagi S Aksoydan B Ekhteiari Salmas R Angeli A. Synthesis, Biological Activity and Multiscale Molecular Modeling Studies for Coumaryl-Carboxamide Derivatives as Selective Carbonic Anhydrase IX Inhibitors. J Enzyme Inhib Med Chem (2017) 32(1):1042–52. doi: 10.1080/14756366.2017.1354857 PMC6009903 28776440

[133] Lin MH Wang JS Hsieh YC Zheng JH Cho EC. NO(2) Functionalized Coumarin Derivatives Suppress Cancer Progression and Facilitate Apoptotic Cell Death in KRAS Mutant Colon Cancer. Chem Biol Interact (2019) 309:108708. doi: 10.1016/j.cbi.2019.06.021 31199928

[134] Yang CS Han SQ Wang X Zhou T Dong XY Bo P. RRLC-DAD-ESI-MS Based and Bioactivity Guided Phytochemical Analysis and Separation of Coumarins From Raw Extracts of Trigonostemon Lutescens. J Pharm BioMed Anal (2019) 169:293–302. doi: 10.1016/j.jpba.2019.02.045 30901623

[135] Lu S Yin S Zhao C Fan L Hu H. Synergistic Anti-Colon Cancer Effect of Glycyrol and Butyrate is Associated With the Enhanced Activation of Caspase-3 and Structural Features of Glycyrol. Food Chem Toxicol (2020) 136:110952. doi: 10.1016/j.fct.2019.110952 31712101

[136] Bisi A Cappadone C Rampa A Farruggia G Sargenti A Belluti F. Coumarin Derivatives as Potential Antitumor Agents: Growth Inhibition, Apoptosis Induction and Multidrug Resistance Reverting Activity. Eur J Med Chem (2017) 127:577–85. doi: 10.1016/j.ejmech.2017.01.020 28109950

[137] Khan S Zafar A Naseem I. Copper-Redox Cycling by Coumarin-Di(2-Picolyl)Amine Hybrid Molecule Leads to ROS-Mediated DNA Damage and Apoptosis: A Mechanism for Cancer Chemoprevention. Chem Biol Interact (2018) 290:64–76. doi: 10.1016/j.cbi.2018.05.010 29803612

[138] Machado TO Cardoso PB Feuser PE Sayer C Araújo PHH. Thiol-Ene Miniemulsion Polymerization of a Biobased Monomer for Biomedical Applications. Colloids Surf B Biointerfaces (2017) 159:509–17. doi: 10.1016/j.colsurfb.2017.07.043 28843199

[139] Bonardi A Falsini M Catarzi D Varano F Di Cesare Mannelli L Tenci B. Structural Investigations on Coumarins Leading to Chromeno[4,3-C]Pyrazol-4-Ones and Pyrano[4,3-C]Pyrazol-4-Ones: New Scaffolds for the Design of the Tumor-Associated Carbonic Anhydrase Isoforms IX and XII. Eur J Med Chem (2018) 146:47–59. doi: 10.1016/j.ejmech.2018.01.033 29407972

[140] Sheng L Yang Y Zhang Y Li N. Chemical Constituents of Patrinia Heterophylla Bunge and Selective Cytotoxicity Against Six Human Tumor Cells. J Ethnopharmacol (2019) 236:129–35. doi: 10.1016/j.jep.2019.03.005 30853646

[141] Sabt A Abdelhafez OM El-Haggar RS Madkour HMF Eldehna WM El-Khrisy E. Novel Coumarin-6-Sulfonamides as Apoptotic Anti-Proliferative Agents: Synthesis, *In Vitro* Biological Evaluation, and QSAR Studies. J Enzyme Inhib Med Chem (2018) 33(1):1095–107. doi: 10.1080/14756366.2018.1477137 PMC6022226 29944015

[142] Ren Y Mu Y Song Y Xie J Yu H Gao S. A New Peptide Ligand for Colon Cancer Targeted Delivery of Micelles. Drug Deliv (2016) 23(5):1763–72. doi: 10.3109/10717544.2015.1077293 26289214

[143] Kang JI Hong JY Choi JS Lee SK. Columbianadin Inhibits Cell Proliferation by Inducing Apoptosis and Necroptosis in HCT116 Colon Cancer Cells. Biomol Ther (Seoul) (2016) 24(3):320–7. doi: 10.4062/biomolther.2015.145 PMC4859796 27098859

[144] Suthiphasilp V Maneerat W Andersen RJ Pyne SG Muanprasat C Seemakhan S. Coumarins and Flavones From the Fruit and Root Extracts of Micromelum Integerrimum. Nat Prod Res (2019) 33(20):2945–50. doi: 10.1080/14786419.2018.1510400 30398372

[145] Salem MA Marzouk MI El-Kazak AM. Synthesis and Characterization of Some New Coumarins With *in Vitro* Antitumor and Antioxidant Activity and High Protective Effects Against DNA Damage. Molecules (2016) 21(2):249. doi: 10.3390/molecules21020249 26907244 PMC6274385

[146] Jantamat P Weerapreeyakul N Puthongking P. Cytotoxicity and Apoptosis Induction of Coumarins and Carbazole Alkaloids From Clausena Harmandiana. Molecules (2019) 24(18). doi: 10.3390/molecules24183385 PMC6767265 31540345

[147] da Cunha MG Rosalen PL Franchin M de Alencar SM Ikegaki M Ransom T. Antiproliferative Constituents of Geopropolis From the Bee Melipona Scutellaris. Planta Med (2016) 82(3):190–4. 10.1055/s-0035-1558142 PMC4792278 26544117

[148] El-Gamal MI Oh CH. Synthesis, *In Vitro* Antiproliferative Activity, and *in Silico* Studies of Fused Tricyclic Coumarin Sulfonate Derivatives. Eur J Med Chem (2014) 84:68–76. doi: 10.1016/j.ejmech.2014.06.064 25016229

[149] Lin MH Cheng CH Chen KC Lee WT Wang YF Xiao CQ. Induction of ROS-Independent JNK-Activation-Mediated Apoptosis by a Novel Coumarin-Derivative, DMAC, in Human Colon Cancer Cells. Chem Biol Interact (2014) 218:42–9. doi: 10.1016/j.cbi.2014.04.015 24812029

[150] Amin KM Eissa AA Abou-Seri SM Awadallah FM Hassan GS. Synthesis and Biological Evaluation of Novel Coumarin-Pyrazoline Hybrids Endowed With Phenylsulfonyl Moiety as Antitumor Agents. Eur J Med Chem (2013) 60:187–98. doi: 10.1016/j.ejmech.2012.12.004 23291120

[151] Tanemossu SA Franke K Arnold N Schmidt J Wabo HK Tane P. Rare Biscoumarin Derivatives and Flavonoids From Hypericum Riparium. Phytochemistry (2014) 105:171–7. doi: 10.1016/j.phytochem.2014.05.008 24930002

[152] Kim AD Han X Piao MJ Hewage SR Hyun CL Cho SJ. Esculetin Induces Death of Human Colon Cancer Cells *via* the Reactive Oxygen Species-Mediated Mitochondrial Apoptosis Pathway. Environ Toxicol Pharmacol (2015) 39(2):982–9. doi: 10.1016/j.etap.2015.03.003 25818986

[153] Paul K Bindal S Luxami V. Synthesis of New Conjugated Coumarin-Benzimidazole Hybrids and Their Anticancer Activity. Bioorg Med Chem Lett (2013) 23(12):3667–72. doi: 10.1016/j.bmcl.2012.12.071 23642480

[154] Lee SY Lim TG Chen H Jung SK Lee HJ Lee MH. Esculetin Suppresses Proliferation of Human Colon Cancer Cells by Directly Targeting β-Catenin. Cancer Prev Res (Phila) (2013) 6(12):1356–64. doi: 10.1158/1940-6207.CAPR-13-0241 PMC3885338 24104353

[155] Xu G Shi C Guo D Wang L Ling Y Han X. Functional-Segregated Coumarin-Containing Telodendrimer Nanocarriers for Efficient Delivery of SN-38 for Colon Cancer Treatment. Acta Biomater (2015) 21:85–98. doi: 10.1016/j.actbio.2015.04.021 25910639 PMC4446163

[156] Rajabi M Hossaini Z Khalilzadeh MA Datta S Halder M Mousa SA. Synthesis of a New Class of Furo[3,2-C]Coumarins and its Anticancer Activity. J Photochem Photobiol B (2015) 148:66–72. doi: 10.1016/j.jphotobiol.2015.03.027 25889947

[157] Kim AD Madduma Hewage SR Piao MJ Kang KA Cho SJ Hyun JW. Esculetin Induces Apoptosis in Human Colon Cancer Cells by Inducing Endoplasmic Reticulum Stress. Cell Biochem Funct (2015) 33(7):487–94. doi: 10.1002/cbf.3146 26439795

[158] Beena T Sudha L Nataraj A Balachandran V Kannan D Ponnuswamy MN. Synthesis, Spectroscopic, Dielectric, Molecular Docking and DFT Studies of (3E)-3-(4-Methylbenzylidene)-3,4-Dihydro-2H-Chromen-2-One: An Anticancer Agent. Chem Cent J (2017) 11:6. doi: 10.1186/s13065-016-0230-8 28119762 PMC5225380

[159] Ercan G Karlitepe A Ozpolat B. Pancreatic Cancer Stem Cells and Therapeutic Approaches. Anticancer Res (2017) 37(6):2761–75. 10.21873/anticanres.11628 28551612

[160] Luchini C Capelli P Scarpa A. Pancreatic Ductal Adenocarcinoma and Its Variants. Surg Pathol Clin (2016) 9(4):547–60. doi: 10.1016/j.path.2016.05.003 27926359

[161] Lu H Niu F Liu F Gao J Sun Y Zhao X. Elevated Glypican-1 Expression is Associated With an Unfavorable Prognosis in Pancreatic Ductal Adenocarcinoma. Cancer Med (2017) 6(6):1181–91. doi: 10.1002/cam4.1064 PMC5463070 28440066

[162] Soppa U Becker W. DYRK Protein Kinases. Curr Biol (2015) 25(12):R488–9. doi: 10.1016/j.cub.2015.02.067 26079075

[163] Fernández-Martínez P Zahonero C Sánchez-Gómez P. DYRK1A: The Double-Edged Kinase as a Protagonist in Cell Growth and Tumorigenesis. Mol Cell Oncol (2015) 2(1):e970048. 27308401 10.4161/23723548.2014.970048 PMC4905233

[164] Jarhad DB Mashelkar KK Kim HR Noh M Jeong LS. Dual-Specificity Tyrosine Phosphorylation-Regulated Kinase 1a (DYRK1A) Inhibitors as Potential Therapeutics. J Med Chem (2018) 61(22):9791–810. doi: 10.1021/acs.jmedchem.8b00185 29985601

[165] Ionescu A Dufrasne F Gelbcke M Jabin I Kiss R Lamoral-Theys D. DYRK1A Kinase Inhibitors With Emphasis on Cancer. Mini Rev Med Chem (2012) 12(13):1315–29. 10.2174/13895575112091315 23016545

[166] Salton M Misteli T. Small Molecule Modulators of Pre-mRNA Splicing in Cancer Therapy. Trends Mol Med (2016) 22(1):28–37. doi: 10.1016/j.molmed.2015.11.005 26700537 PMC4707101

[167] Seifert A Allan LA Clarke PR. DYRK1A Phosphorylates Caspase 9 at an Inhibitory Site and is Potently Inhibited in Human Cells by Harmine. FEBS J (2008) 275(24):6268–80. doi: 10.1111/j.1742-4658.2008.06751.x 19016842

[168] Luna J Boni J Cuatrecasas M Bofill-De Ros X Núñez-Manchón E Gironella M. DYRK1A Modulates C-MET in Pancreatic Ductal Adenocarcinoma to Drive Tumour Growth. Gut (2019) 68(8):1465–76. doi: 10.1136/gutjnl-2018-316128 30343272

[169] Zhao C Wang D Gao Z Kan H Qiu F Chen L. Licocoumarone Induces BxPC-3 Pancreatic Adenocarcinoma Cell Death by Inhibiting DYRK1A. Chem Biol Interact (2020) 316:108913. doi: 10.1016/j.cbi.2019.108913 31838052

[170] Fu Y Tan L Meng L Lei X. Therapeutic Effects of Paclitaxel Loaded Polyethylene Glycol-Polylactic Acid-Glycolic Acid Copolymer Nanoparticles on Pancreatic Cancer in Rats. J Nanosci Nanotechnol (2020) 20(12):7271–5. doi: 10.1166/jnn.2020.18608 32711590

[171] Kalpana K Priyadarshini E Sreeja S Jagan K Anuradha CV. Scopoletin Intervention in Pancreatic Endoplasmic Reticulum Stress Induced by Lipotoxicity. Cell Stress Chaperones (2018) 23(5):857–69. doi: 10.1007/s12192-018-0893-2 PMC6111101 29574508

[172] Kahveci B Yılmaz F Menteşe E. Design, Synthesis, and Biological Evaluation of Coumarin-Triazole Hybrid Molecules as Potential Antitumor and Pancreatic Lipase Agents. Arch Pharm (Weinheim) (2017) 350(8). doi: 10.1002/ardp.201600369 28543820

[173] Mattarei A Romio M Managò A Zoratti M Paradisi C Szabò I. Novel Mitochondria-Targeted Furocoumarin Derivatives as Possible Anti-Cancer Agents. Int J Cancer (2018) 8:122. doi: 10.3389/fonc.2018.00122 PMC5925966 29740538

[174] Jun M Bacay AF Moyer J Webb A Carrico-Moniz D. Synthesis and Biological Evaluation of Isoprenylated Coumarins as Potential Anti-Pancreatic Cancer Agents. Bioorg Med Chem Lett (2014) 24(19):4654–8. doi: 10.1016/j.bmcl.2014.08.038 25205194

[175] Nasr T Bondock S Rashed HM Fayad W Youns M Sakr TM. Novel Hydrazide-Hydrazone and Amide Substituted Coumarin Derivatives: Synthesis, Cytotoxicity Screening, Microarray, Radiolabeling and *In Vivo* Pharmacokinetic Studies. Eur J Med Chem (2018) 151:723–39. doi: 10.1016/j.ejmech.2018.04.014 29665526

[176] Vinayagam R Xu B. 7, 8-Dihydroxycoumarin (Daphnetin) Protects INS-1 Pancreatic β-Cells Against Streptozotocin-Induced Apoptosis. Phytomedicine (2017) 24:119–26. doi: 10.1016/j.phymed.2016.11.023 28160851

[177] Musa MA Gbadebo AJ Latinwo LM Badisa VL. 7,8-Dihydroxy-3-(4-Nitrophenyl)Coumarin Induces Cell Death *via* Reactive Oxygen Species-Independent S-Phase Cell Arrest. J Biochem Mol Toxicol (2018) 32(12):e22203. doi: 10.1002/jbt.22203 30368977

[178] Devji T Reddy C Woo C Awale S Kadota S Carrico-Moniz D. Pancreatic Anticancer Activity of a Novel Geranylgeranylated Coumarin Derivative. Bioorg Med Chem Lett (2011) 21(19):5770–3. doi: 10.1016/j.bmcl.2011.08.005 21880488

[179] Arora R Sawney S Saini V Steffi C Tiwari M Saluja D. Esculetin Induces Antiproliferative and Apoptotic Response in Pancreatic Cancer Cells by Directly Binding to KEAP1. Mol Cancer (2016) 15(1):64. doi: 10.1186/s12943-016-0550-2 27756327 PMC5069780

[180] Sun S Phrutivorapongkul A Dibwe DF Balachandran C Awale S. Chemical Constituents of Thai Citrus Hystrix and Their Antiausterity Activity Against the PANC-1 Human Pancreatic Cancer Cell Line. (2018) 81(8):1877–83. doi: 10.1021/acs.jnatprod.8b00405 30070833

[181] Morohashi H Kon A Nakai M Yamaguchi M Kakizaki I Yoshihara S. Study of Hyaluronan Synthase Inhibitor, 4-Methylumbelliferone Derivatives on Human Pancreatic Cancer Cell (KP1-Nl). Biochem Biophys Res Commun (2006) 345(4):1454–9. doi: 10.1016/j.bbrc.2006.05.037 16730656

[182] Farley CM Dibwe DF Ueda JY Hall EA Awale S Magolan J. Evaluation of Synthetic Coumarins for Antiausterity Cytotoxicity Against Pancreatic Cancers. Bioorg Med Chem Lett (2016) 26(5):1471–4. doi: 10.1016/j.bmcl.2016.01.054 26832787

[183] Awale S Okada T Dibwe DF Maruyama T Takahara S Okada T. Design and Synthesis of Functionalized Coumarins as Potential Anti-Austerity Agents That Eliminates Cancer Cells’ Tolerance to Nutrition Starvation. J Nat Prod (2019) 29(14):1779–84. 10.1016/j.bmcl.2019.05.010 31097375

[184] Nasr T Bondock S Youns M. Anticancer Activity of New Coumarin Substituted Hydrazide-Hydrazone Derivatives. Eur J Med Chem (2014) 76:539–48. doi: 10.1016/j.ejmech.2014.02.026 24607878

[185] Liu Z Liu J Zhao K Shi Q Zuo T Wang G. Role of Daphnetin in Rat Severe Acute Pancreatitis Through the Regulation of TLR4/NF-[Formula: See Text]B Signaling Pathway Activation. Am J Chin Med (2016) 44(1):149–63. doi: 10.1142/S0192415X16500105 26916920

[186] Ma S Chan KW Guan XY. In Search of Liver Cancer Stem Cells. Stem Cell Rev (2008) 4(3):179–92. doi: 10.1007/s12015-008-9035-z 18663610

[187] Tomuleasa C Soritau O Rus-Ciuca D Pop T Todea D Mosteanu O. Isolation and Characterization of Hepatic Cancer Cells With Stem-Like Properties From Hepatocellular Carcinoma. J Gastrointestin Liver Dis (2010) 19(1):61–7. 20361077

[188] Lee TK Castilho A Ma S Ng IO. Liver Cancer Stem Cells: Implications for a New Therapeutic Target. Liver Int (2009) 29(7):955–65. doi: 10.1111/j.1478-3231.2009.02040.x 19490415

[189] Chuang WL Su CC Lin PY Lin CC Chen YL. Sann-Joong-Kuey-Jian-Tang Induces Autophagy in HepG2 Cells *via* Regulation of the Phosphoinositide-3 Kinase/Akt/mammalian Target of Rapamycin and P38 Mitogen-Activated Protein Kinase Pathways. Mol Med Rep (2015) 12(2):1677–84. doi: 10.3892/mmr.2015.3573 PMC4464480 25847489

[190] Li N Zheng D Xue J Guo W Shi J Sun J. Cidan Inhibits Liver Cancer Cell Growth by Reducing COX-2 and VEGF Expression and Cell Cycle Arrest. Exp Ther Med (2015) 9(5):1709–18. doi: 10.3892/etm.2015.2351 PMC4471759 26136881

[191] He G Cao X He M Sheng X Wu Y Ai X. Casticin Inhibits Self-Renewal of Liver Cancer Stem Cells From the MHCC97 Cell Line. Oncol Lett (2014) 7(6):2023–8. doi: 10.3892/ol.2014.1972 PMC4049684 24932283

[192] Levine B Klionsky DJ. Development by Self-Digestion: Molecular Mechanisms and Biological Functions of Autophagy. Dev Cell (2004) 6(4):463–77. doi: 10.1016/S1534-5807(04)00099-1 15068787

[193] Kondo Y Kanzawa T Sawaya R Kondo S. The Role of Autophagy in Cancer Development and Response to Therapy. Nat Rev Cancer (2005) 5(9):726–34. doi: 10.1038/nrc1692 16148885

[194] Yorimitsu T Klionsky DJ. Autophagy: Molecular Machinery for Self-Eating. Cell Death Differ (2005) 12(Suppl 2):1542–52. doi: 10.1038/sj.cdd.4401765 PMC1828868 16247502

[195] Kung CP Budina A Balaburski G Bergenstock MK Murphy M. Autophagy in Tumor Suppression and Cancer Therapy. Crit Rev Eukaryot Gene Expr (2011) 21(1):71–100. doi: 10.1615/CritRevEukarGeneExpr.v21.i1.50 21967333 PMC3187613

[196] Thorburn A. Apoptosis and Autophagy: Regulatory Connections Between Two Supposedly Different Processes. Apoptosis (2008) 13(1):1–9. doi: 10.1007/s10495-007-0154-9 17990121 PMC2601595

[197] Cui X Qin X. Hydroxypyridinone-Coumarin Inhibits the Proliferation of MHCC97 and HepG2 Human Hepatocellular Carcinoma Cells and Down-Regulates the Phosphoinositide-3 Kinase Pathway. Med Sci Monit (2020) 26:e920785. doi: 10.12659/MSM.920785 32218414 PMC7133445

[198] Salar U Taha M Khan KM Ismail NH Imran S Perveen S. Syntheses of New 3-Thiazolyl Coumarin Derivatives, *In Vitro* α-Glucosidase Inhibitory Activity, and Molecular Modeling Studies. Eur J Med Chem (2016) 122:196–204. doi: 10.1016/j.ejmech.2016.06.037 27371923

[199] Taha M Ismail NH Lalani S Fatmi MQ Atia Tul W Siddiqui S. Synthesis of Novel Inhibitors of α-Glucosidase Based on the Benzothiazole Skeleton Containing Benzohydrazide Moiety and Their Molecular Docking Studies. Eur J Med Chem (2015) 92:387–400. doi: 10.1016/j.ejmech.2015.01.009 25585009

[200] Taha M Ismail NH Imran S Rokei MQB Saad SM Khan KM. Synthesis of New Oxadiazole Derivatives as α-Glucosidase Inhibitors. Bioorg Med Chem (2015) 23(15):4155–62. doi: 10.1016/j.bmc.2015.06.060 26183542

[201] Imran S Taha M Ismail NH Kashif SM Rahim F Jamil W. Synthesis of Novel Flavone Hydrazones: *in-Vitro* Evaluation of α-Glucosidase Inhibition, QSAR Analysis and Docking Studies. Eur J Med Chem (2015) 105:156–70. doi: 10.1016/j.ejmech.2015.10.017 26491979

[202] Taha M Ismail NH Imran S Selvaraj M Rahim F. Synthesis of Novel Inhibitors of β-Glucuronidase Based on the Benzothiazole Skeleton and Their Molecular Docking Studies. RSC Adv (2016) 6(4):3003–12. doi: 10.1039/C5RA23072A

[203] Taha M Sultan S Nuzar HA Rahim F Imran S Ismail NH. Synthesis and Biological Evaluation of Novel N-Arylidenequinoline-3-Carbohydrazides as Potent β-Glucuronidase Inhibitors. Bioorg Med Chem (2016) 24(16):3696–704. doi: 10.1016/j.bmc.2016.06.008 27312423

[204] Salum LB Mascarello A Canevarolo RR Altei WF Laranjeira AB Neuenfeldt PD. N-(1’-Naphthyl)-3,4,5-Trimethoxybenzohydrazide as Microtubule Destabilizer: Synthesis, Cytotoxicity, Inhibition of Cell Migration and *In Vivo* Activity Against Acute Lymphoblastic Leukemia. Eur J Med Chem (2015) 96:504–18. doi: 10.1016/j.ejmech.2015.02.041 25951294

[205] Bayomi SM Moustafa MA Maarouf AR Abutaleb MH. Design, Synthesis, Biological Activity and Molecular Modeling of New Heterocyclic Tetrazole Derivatives. J Am Sci (2016) 12(1):40–56.

[206] Nagamallu R Srinivasan B Ningappa MB Kariyappa AK. Synthesis of Novel Coumarin Appended Bis(Formylpyrazole) Derivatives: Studies on Their Antimicrobial and Antioxidant Activities. Bioorg Med Chem Lett (2016) 26(2):690–4. doi: 10.1016/j.bmcl.2015.11.038 26631319

[207] Refat HM Fadda AA. Synthesis and Antimicrobial Activity of Some Novel Hydrazide, Benzochromenone, Dihydropyridine, Pyrrole, Thiazole and Thiophene Derivatives. Eur J Med Chem (2013) 70:419–26. doi: 10.1016/j.ejmech.2013.09.003 24184775

[208] Ledour G Moroy G Rouffet M Bourguet E Guillaume D Decarme M. Introduction of the 4-(4-Bromophenyl)Benzenesulfonyl Group to Hydrazide Analogs of Ilomastat Leads to Potent Gelatinase B (MMP-9) Inhibitors With Improved Selectivity. Bioorg Med Chem (2008) 16(18):8745–59. doi: 10.1016/j.bmc.2008.07.041 18782669

[209] Yang L Wang P Wu JF Yang LM Wang RR Pang W. Design, Synthesis and Anti-HIV-1 Evaluation of Hydrazide-Based Peptidomimetics as Selective Gelatinase Inhibitors. Bioorg Med Chem (2016) 24(9):2125–36. doi: 10.1016/j.bmc.2016.03.043 27039251

[210] Cui X Qin X. Hydroxypyridinone-Coumarin Inhibits the Proliferation of MHCC97 and HepG2 Human Hepatocellular Carcinoma Cells and Down-Regulates the Phosphoinositide-3 Kinase Pathway. J Agric Food Chem (2020) 26:e920785. doi: 10.12659/MSM.920785 PMC7133445 32218414

[211] Thati B Noble A Creaven BS Walsh M McCann M Kavanagh K. *In Vitro* Anti-Tumour and Cyto-Selective Effects of Coumarin-3-Carboxylic Acid and Three of its Hydroxylated Derivatives, Along With Their Silver-Based Complexes, Using Human Epithelial Carcinoma Cell Lines. Molecules (2007) 248(2):321–31. doi: 10.1016/j.canlet.2006.08.009 16996681

[212] Wang J Lu ML Dai HL Zhang SP Wang HX Wei N. Esculetin, a Coumarin Derivative, Exerts *In Vitro* and *In Vivo* Antiproliferative Activity Against Hepatocellular Carcinoma by Initiating a Mitochondrial-Dependent Apoptosis Pathway. Braz J Med Biol Res (2015) 48(3):245–53. doi: 10.1590/1414-431x20144074 PMC4381945 25517918

[213] Zhang L Jiang G Yao F He Y Liang G Zhang Y. Growth Inhibition and Apoptosis Induced by Osthole, a Natural Coumarin, in Hepatocellular Carcinoma. PloS One (2012) 7(5):e37865. doi: 10.1371/journal.pone.0037865 22662241 PMC3360675

[214] Thati B Noble A Creaven BS Walsh M McCann M Devereux M. Role of Cell Cycle Events and Apoptosis in Mediating the Anti-Cancer Activity of a Silver(I) Complex of 4-Hydroxy-3-Nitro-Coumarin-Bis(Phenanthroline) in Human Malignant Cancer Cells. Eur J Pharmacol (2009) 602(2-3):203–14. doi: 10.1016/j.ejphar.2008.11.020 19041861

[215] Thati B Noble A Creaven BS Walsh M Kavanagh K Egan DA. An *In Vitro* Investigation of the Induction of Apoptosis and Modulation of Cell Cycle Events in Human Cancer Cells by Bisphenanthroline-Coumarin-6,7-Dioxacetatocopper(II) Complex. Chem Biol Interact (2007) 168(2):143–58. doi: 10.1016/j.cbi.2007.04.003 17512508

[216] Neelgundmath M Dinesh KR Mohan CD Li F Dai X Siveen KS. Novel Synthetic Coumarins That Targets NF-κB in Hepatocellular Carcinoma. Bioorg Med Chem Lett (2015) 25(4):893–7. doi: 10.1016/j.bmcl.2014.12.065 25592709

[217] Okamoto T Kobayashi T Yoshida S. Chemical Aspects of Coumarin Compounds for the Prevention of Hepatocellular Carcinomas. Curr Med Chem Anticancer Agents (2005) 5(1):47–51. doi: 10.2174/1568011053352622 15720260

[218] Jantamat P Weerapreeyakul N. Cytotoxicity and Apoptosis Induction of Coumarins and Carbazole Alkaloids From Clausena Harmandiana. Molecules (2019) 24(18):3385. doi: 10.3390/molecules24183385 PMC6767265 31540345

[219] Goud NS Pooladanda V Mahammad GS Jakkula P Gatreddi S Qureshi IA. Synthesis and Biological Evaluation of Morpholines Linked Coumarin-Triazole Hybrids as Anticancer Agents. (2019) 94(5):1919–29. doi: 10.1111/cbdd.13578 31169963

[220] Mohamed TK Batran RZ Elseginy SA Ali MM Mahmoud AE. Synthesis, Anticancer Effect and Molecular Modeling of New Thiazolylpyrazolyl Coumarin Derivatives Targeting VEGFR-2 Kinase and Inducing Cell Cycle Arrest and Apoptosis. Bioorg Chem (2019) 85:253–73. doi: 10.1016/j.bioorg.2018.12.040 30641320

[221] Elshemy HAH Zaki MA. Design and Synthesis of New Coumarin Hybrids and Insight Into Their Mode of Antiproliferative Action. Bioorg Med Chem (2017) 25(3):1066–75. doi: 10.1016/j.bmc.2016.12.019 28038941

[222] Yamazaki T Tokiwa T. Isofraxidin, a Coumarin Component From Acanthopanax Senticosus, Inhibits Matrix Metalloproteinase-7 Expression and Cell Invasion of Human Hepatoma Cells. Biol Pharm Bull (2010) 33(10):1716–22. doi: 10.1248/bpb.33.1716 20930381

[223] Lou LL Zhao P Cheng ZY Guo R Yao GD Wang XB. A New Coumarin From Juglans Mandshurica Maxim Induce Apoptosis in Hepatocarcinoma Cells. Nat Prod Res (2019) 33(12):1791–3. doi: 10.1080/14786419.2018.1434646 29397774

[224] Pan J Zhang Q Zhao CY Zheng RL. Redifferentiation of Human Hepatoma Cells Induced by Synthesized Coumarin. Cell Biol Int (2004) 28(5):329–33. doi: 10.1016/j.cellbi.2004.02.002 15193276

[225] Torre LA Bray F Siegel RL Ferlay J Lortet-Tieulent J Jemal A. Global Cancer Statistics, 2012. CA Cancer J Clin (2015) 65(2):87–108. doi: 10.3322/caac.21262 25651787

[226] Lim B Jiang Y. Current and Emerging Systemic Therapy in Gastro-Esophageal Cancer "The Old and New Therapy for Metastatic Disease, The Role of Adjuvant and Neoadjuvant Therapy for Localized Disease". Curr Clin Pharmacol (2015) 10(4):267–78. doi: 10.2174/1574884710666151020100329 26548904

[227] Facompre N Nakagawa H Herlyn M Basu D. Stem-Like Cells and Therapy Resistance in Squamous Cell Carcinomas. Adv Pharmacol (2012) 65:235–65. doi: 10.1016/B978-0-12-397927-8.00008-7 PMC3595160 22959028

[228] Rassouli FB Matin MM Saeinasab M. Cancer Stem Cells in Human Digestive Tract Malignancies. Tumour Biol (2016) 37(1):7–21. doi: 10.1007/s13277-015-4155-y 26446457

[229] Rassouli FB Matin MM Bahrami AR Ghaffarzadegan K Cheshomi H Lari S. Evaluating Stem and Cancerous Biomarkers in CD15+CD44+ KYSE30 Cells. Tumour Biol (2013) 34(5):2909–20. doi: 10.1007/s13277-013-0853-5 23797812

[230] Lu C Xu F Gu J Yuan Y Zhao G Yu X. Clinical and Biological Significance of Stem-Like CD133(+)CXCR4(+) Cells in Esophageal Squamous Cell Carcinoma. J Thorac Cardiovasc Surg (2015) 150(2):386–95. doi: 10.1016/j.jtcvs.2015.05.030 26092504

[231] Tang KH Dai YD Tong M Chan YP Kwan PS Fu L. A CD90(+) Tumor-Initiating Cell Population With an Aggressive Signature and Metastatic Capacity in Esophageal Cancer. Cancer Res (2013) 73(7):2322–32. doi: 10.1158/0008-5472.CAN-12-2991 23382045

[232] Genovese S Epifano F. Auraptene: A Natural Biologically Active Compound With Multiple Targets. Curr Drug Targets (2011) 12(3):381–6. doi: 10.2174/138945011794815248 20955144

[233] Tanaka T Kawabata K Kakumoto M Matsunaga K Mori H Murakami A. Chemoprevention of 4-Nitroquinoline 1-Oxide-Induced Oral Carcinogenesis by Citrus Auraptene in Rats. Carcinogenesis (1998) 19(3):425–31. doi: 10.1093/carcin/19.3.425 9525276

[234] Hayashi K Suzuki R Miyamoto S Shin-Ichiroh Y Kohno H Sugie S. Citrus Auraptene Suppresses Azoxymethane-Induced Colonic Preneoplastic Lesions in C57BL/KsJ-db/db Mice. Nutr Cancer (2007) 58(1):75–84. doi: 10.1080/01635580701308216 17571970

[235] Kohno H Suzuki R Curini M Epifano F Maltese F Gonzales SP. Dietary Administration With Prenyloxycoumarins, Auraptene and Collinin, Inhibits Colitis-Related Colon Carcinogenesis in Mice. Int J Cancer (2006) 118(12):2936–42. doi: 10.1002/ijc.21719 16395701

[236] Tanaka T de Azevedo MB Durán N Alderete JB Epifano F Genovese S. Colorectal Cancer Chemoprevention by 2 Beta-Cyclodextrin Inclusion Compounds of Auraptene and 4’-Geranyloxyferulic Acid. Int J Cancer (2010) 126(4):830–40. doi: 10.1002/ijc.24833 19688830

[237] Kawabata K Tanaka T Yamamoto T Ushida J Hara A Murakami A. Suppression of N-Nitrosomethylbenzylamine-Induced Rat Esophageal Tumorigenesis by Dietary Feeding of 1’-Acetoxychavicol Acetate. Jpn J Cancer Res (2000) 91(2):148–55. doi: 10.1111/j.1349-7006.2000.tb00926.x PMC5926320 10761701

[238] Sakata K Hara A Hirose Y Yamada Y Kuno T Katayama M. Dietary Supplementation of the Citrus Antioxidant Auraptene Inhibits N,N-Diethylnitrosamine-Induced Rat Hepatocarcinogenesis. Oncology (2004) 66(3):244–52. doi: 10.1159/000078001 15218316

[239] Hara A Sakata K Yamada Y Kuno T Kitaori N Oyama T. Suppression of Beta-Catenin Mutation by Dietary Exposure of Auraptene, a Citrus Antioxidant, in N,N-Diethylnitrosamine-Induced Hepatocellular Carcinomas in Rats. Oncol Rep (2005) 14(2):345–51. 16012713

[240] Krishnan P Yan KJ Windler D Tubbs J Grand R Li BD. Citrus Auraptene Suppresses Cyclin D1 and Significantly Delays N-Methyl Nitrosourea Induced Mammary Carcinogenesis in Female Sprague-Dawley Rats. BMC Cancer (2009) 9:259. doi: 10.1186/1471-2407-9-259 19640308 PMC2724550

[241] Tang M Ogawa K Asamoto M Hokaiwado N Seeni A Suzuki S. Protective Effects of Citrus Nobiletin and Auraptene in Transgenic Rats Developing Adenocarcinoma of the Prostate (TRAP) and Human Prostate Carcinoma Cells. Cancer Sci (2007) 98(4):471–7. doi: 10.1111/j.1349-7006.2007.00417.x PMC11158555 17284254

[242] Murakami A Kuki W Takahashi Y Yonei H Nakamura Y Ohto Y. Auraptene, a Citrus Coumarin, Inhibits 12-O-Tetradecanoylphorbol-13-Acetate-Induced Tumor Promotion in ICR Mouse Skin, Possibly Through Suppression of Superoxide Generation in Leukocytes. Jpn J Cancer Res (1997) 88(5):443–52. doi: 10.1111/j.1349-7006.1997.tb00402.x PMC5921462 9247600

[243] Mori H Niwa K Zheng Q Yamada Y Sakata K Yoshimi N. Cell Proliferation in Cancer Prevention; Effects of Preventive Agents on Estrogen-Related Endometrial Carcinogenesis Model and on an In Vitro Model in Human Colorectal Cells. Mutat Res (2001) 480-481:201–7. doi: 10.1016/S0027-5107(01)00200-7 11506814

[244] Moon JY Kim H Cho SK. Auraptene, a Major Compound of Supercritical Fluid Extract of Phalsak (Citrus Hassaku Hort Ex Tanaka), Induces Apoptosis Through the Suppression of mTOR Pathways in Human Gastric Cancer SNU-1 Cells. Evid Based Complement Alternat Med (2015) 2015:402385. doi: 10.1155/2015/402385 26351512 PMC4550746

[245] Jun DY Kim JS Park HS Han CR Fang Z Woo MH. Apoptogenic Activity of Auraptene of Zanthoxylum Schinifolium Toward Human Acute Leukemia Jurkat T Cells is Associated With ER Stress-Mediated Caspase-8 Activation That Stimulates Mitochondria-Dependent or -Independent Caspase Cascade. Carcinogenesis (2007) 28(6):1303–13. doi: 10.1093/carcin/bgm028 17301064

[246] Jang Y Han J Kim SJ Kim J Lee MJ Jeong S. Suppression of Mitochondrial Respiration With Auraptene Inhibits the Progression of Renal Cell Carcinoma: Involvement of HIF-1α Degradation. Oncotarget (2015) 6(35):38127–38. doi: 10.18632/oncotarget.5511 PMC4741988 26474388

[247] Krishnan P Kleiner-Hancock H. Effects of Auraptene on IGF-1 Stimulated Cell Cycle Progression in the Human Breast Cancer Cell Line, MCF-7. Int J Breast Cancer (2012) 2012:502092. doi: 10.1155/2012/502092 23320178 PMC3536038

[248] Epifano F Genovese S Miller R Majumdar AP. Auraptene and its Effects on the Re-Emergence of Colon Cancer Stem Cells. Phytother Res (2013) 27(5):784–6. doi: 10.1002/ptr.4773 PMC3496036 22761031

[249] Saboor-Maleki S Rassouli FB Matin MM Iranshahi M. Auraptene Attenuates Malignant Properties of Esophageal Stem-Like Cancer Cells. Technol Cancer Res Treat (2017) 16(4):519–27. doi: 10.1177/1533034616650119 PMC5616061 27207438

[250] Zhang ZR Leung WN Cheung HY Chan CW. Osthole: A Review on Its Bioactivities, Pharmacological Properties, and Potential as Alternative Medicine. Evid Based Complement Alternat Med (2015) 2015:919616. doi: 10.1155/2015/919616 26246843 PMC4515521

[251] Zhang W Ma D Zhao Q Ishida T. The Effect of the Major Components of Fructus Cnidii on Osteoblasts In Vitro. J Acupunct Meridian Stud (2010) 3(1):32–7. doi: 10.1016/S2005-2901(10)60005-2 20633513

[252] Ko FN Wu TS Liou MJ Huang TF Teng CM. Vasorelaxation of Rat Thoracic Aorta Caused by Osthole Isolated From Angelica Pubescens. Eur J Pharmacol (1992) 219(1):29–34. doi: 10.1016/0014-2999(92)90576-P 1327835

[253] Zhang Q Qin L He W Van Puyvelde L Maes D Adams A. Coumarins From Cnidium Monnieri and Their Antiosteoporotic Activity. Planta Med (2007) 73(1):13–9. doi: 10.1055/s-2006-951724 17315308

[254] Zimecki M Artym J Cisowski W Mazol I Włodarczyk M Gleńsk M. Immunomodulatory and Anti-Inflammatory Activity of Selected Osthole Derivatives. Z Naturforsch C J Biosci (2009) 64(5-6):361–8. doi: 10.1515/znc-2009-5-610 19678539

[255] Okamoto T Yoshida S Kobayashi T Okabe S. Inhibition of Concanavalin A-Induced Mice Hepatitis by Coumarin Derivatives. Jpn J Pharmacol (2001) 85(1):95–7. doi: 10.1254/jjp.85.95 11243581

[256] Matsuda H Tomohiro N Ido Y Kubo M. Anti-Allergic Effects of Cnidii Monnieri Fructus (Dried Fruits of Cnidium Monnieri) and its Major Component, Osthol. Biol Pharm Bull (2002) 25(6):809–12. doi: 10.1248/bpb.25.809 12081154

[257] Luszczki JJ Andres-Mach M Cisowski W Mazol I Glowniak K Czuczwar SJ. Osthole Suppresses Seizures in the Mouse Maximal Electroshock Seizure Model. Eur J Pharmacol (2009) 607(1-3):107–9. doi: 10.1016/j.ejphar.2009.02.022 19236860

[258] Xu X Zhang Y Qu D Jiang T Li S. Osthole Induces G2/M Arrest and Apoptosis in Lung Cancer A549 Cells by Modulating PI3K/Akt Pathway. J Exp Clin Cancer Res (2011) 30(1):33. doi: 10.1186/1756-9966-30-33 21447176 PMC3073874

[259] Chou SY Hsu CS Wang KT Wang MC Wang CC. Antitumor Effects of Osthol From Cnidium Monnieri: An *In Vitro* and *In Vivo Study* . Phytother Res (2007) 21(3):226–30. doi: 10.1002/ptr.2044 17154232

[260] Feng H Lu JJ Wang Y Pei L Chen X. Osthole Inhibited TGF β-Induced Epithelial-Mesenchymal Transition (EMT) by Suppressing NF-κB Mediated Snail Activation in Lung Cancer A549 Cells. Cell Adh Migr (2017) 11(5-6):464–75. doi: 10.1080/19336918.2016.1259058 PMC5810511 28146373

[261] Zhu X Li Z Li T Long F Lv Y Liu L. Osthole Inhibits the PI3K/AKT Signaling Pathway via Activation of PTEN and Induces Cell Cycle Arrest and Apoptosis in Esophageal Squamous Cell Carcinoma. BioMed Pharmacother (2018) 102:502–9. doi: 10.1016/j.biopha.2018.03.106 29579711

